# Prediction of stock price movement using an improved NSGA-II-RF algorithm with a three-stage feature engineering process

**DOI:** 10.1371/journal.pone.0287754

**Published:** 2023-06-28

**Authors:** Xiaohua Zeng, Jieping Cai, Changzhou Liang, Chiping Yuan

**Affiliations:** 1 School of Economics and Trade, Guangzhou Xinhua University, Dongguan, China; 2 Lingnan College, Sun Yat-Sen University, Guangzhou, China; Universidad de Guadalajara, MEXICO

## Abstract

Prediction of stock price has been a hot topic in artificial intelligence field. Computational intelligent methods such as machine learning or deep learning are explored in the prediction system in recent years. However, making accurate predictions of stock price direction is still a big challenge because stock prices are affected by nonlinear, nonstationary, and high dimensional features. In previous works, feature engineering was overlooked. How to select the optimal feature sets that affect stock price is a prominent solution. Hence, our motivation for this article is to propose an improved many-objective optimization algorithm integrating random forest (I-NSGA-II-RF) algorithm with a three-stage feature engineering process in order to decrease the computational complexity and improve the accuracy of prediction system. Maximizing accuracy and minimizing the optimal solution set are the optimization directions of the model in this study. The integrated information initialization population of two filtered feature selection methods is used to optimize the I-NSGA-II algorithm, using multiple chromosome hybrid coding to synchronously select features and optimize model parameters. Finally, the selected feature subset and parameters are input to the RF for training, prediction, and iterative optimization. Experimental results show that the I-NSGA-II-RF algorithm has the highest average accuracy, the smallest optimal solution set, and the shortest running time compared to the unmodified multi-objective feature selection algorithm and the single target feature selection algorithm. Compared to the deep learning model, this model has interpretability, higher accuracy, and less running time.

## 1. Introduction

Stock market forecasting is one of the most challenging research topics in the financial field. However, it is difficult to build a model or system that can predict the direction of stock prices with optimal accuracy because stock prices have typical characteristics of nonlinearity, high noise, and dynamic change [1–3]. In recent years, with the development of AI technologies, many researchers have applied machine learning (ML) and deep learning (DL) models to forecast stock prices. ML methods include decision trees, back propagation (BP) neural networks, genetic algorithms (GA), support vector machines (SVM), and random forest (RF) [4, 5]. DL methods include recurrent neural networks (RNN), convolutional neural networks (CNN), feedforward neural networks (FFNN) and so on [6]. Multi-objective optimization algorithms such as Non-dominated Sorting Genetic Algorithm (NSGA-II) also have been widely used in many practical fields [7–10].

Kara et al. studied the prediction performance of SVM model and BP neural network in stock market. The study showed BP model achieved higher accuracy than that of the SVM model [11]. Krauss et al. compared RF, DNN, and gradient-boosted trees models in forecasting stock prices [12]. Experiment results indicated that RF had the best performance. Basak et al. applied two tree-based classifiers, namely RF and extreme gradient boosting (XGBoost) to predict stock price direction. The experimental results showed the tree-based models had the higher accuracy compared with SVM, Logistic Regression, and ANN models [1, 13]. Ampomah et al. proposed tree-based ensemble classifiers to forecast stock price movement [14]. RNNs also have been widely proposed by expert researchers [15]. Long short-term memory (LSTM), a successful variant of RNN, has been proven to be a prominent model in forecasting financial time series [16].

Just as DL methods, XGBoost has been proven as an outstanding algorithm in numerous machine learning since its initial introduction in 2014 [17]. Although ML and DL models have achieved superior performances, many studies propose ML or DL models do not consider the importance of feature engineering to improve the accuracy of the prediction model [18]. As mentioned above, complex and high dimensional features affect stock price, feature engineering is necessary as it enables prediction algorithms to select optimal feature sets to improve computational efficiency [17]. Feature engineering architecture in a stock price movement prediction is mainly composed of a feature set expansion module, a classifier module and a feature selection module. According to different feature subset evaluation strategies, feature selection methods can be roughly divided into two categories: filter method and wrapper method [19]. The filter method selects features based on their statistical characteristics, which has fast calculation speed and low accuracy. The wrapper method uses a hybrid classifier to select the optimal feature subset, which has high accuracy and low computational efficiency.

In recent years, there are a number of effective multi-objective approaches designed for optimizing feature engineering. Being motivated by this, we propose an improved many-objective optimization algorithm integrating random forest (I-NSGA-II-RF) algorithm with a three-stage feature engineering process. The contributions of our study are as follows. (1) Stock price direction prediction is a complex nonlinear problem that requires considering various features and factors, as well as their interactions and influences. Therefore, an algorithm that can simultaneously optimize multiple objectives, namely maximizing classification accuracy and minimizing feature quantity, is needed. (2) The I-NSGA-II-RF algorithm is a hybrid algorithm based on multi-objective genetic algorithm and random forest, which can effectively search the high-dimensional feature space, find the optimal or near-optimal feature subsets, and simultaneously optimize the key parameters of random forest, improving prediction performance and reducing computational complexity. (3) The I-NSGA-II-RF algorithm also adopts some improvement strategies, such as combining filtering and wrapping methods, hybrid initialization, external archive mechanism, etc., to improve the convergence speed, diversity and quality of solutions.

The novelty of the model proposed in this article is as follows. Firstly, the original stock price datasets are denoised and technical indicators are generated through a three-stage feature engineering approach. Secondly, the model proposed in this article combines the advantages of two types of feature selection methods and proposes a stock prediction system that balances efficiency and performance. The NSGA-II-RF algorithm proposed in this article is based on the wrapper method, which improves the performance of the classifier by removing irrelevant redundant features and selecting feature subsets to improve the classification performance of the classifier. In the I-NSGA-II initialization stage of the algorithm, two filtering methods is combined to preprocess the selection of feature populations, further improving the efficiency of the algorithm. Thirdly, the stock forecasting model is constructed by combining the multi-objective optimization (NSGA-II) algorithm and the random forest algorithm (RF) to select features and predict the change direction of the stock.

The rest of this paper is organized as follows. Section 2 introduces related work. Section 3 describes the methodology and the proposed stock price direction prediction system. Section 4 provides experimental results and comparison. Section 5 concludes this study.

## 2. Related work

In the past decade, there have been multiple methodologies to predict stock prices such as computational intelligent methods. However, computational intelligent methods still need to improve the prediction efficiency. As the stock price data are high-noisy and nonlinear, some studies focus on feature engineering to enhance the model performance in computational intelligent methods.

Feature selection methods have their own advantages as well as disadvantages. Filter method is independent of the classifier and uses some specific evaluation criteria to evaluate the features. The evaluation criteria include the statistical methods [20, 21] (e.g., Student’s t-test and Chi-square test), the information theory-based methods [20] (e.g., Entropy and Kullback-Leibler divergence), other search techniques [22] (e.g., the correlation-based feature selection algorithm) and Markov blanket filter [23]. The filter methods are simple and efficient. However, they may remove relevant features for a particular class label with fewer instances, so the classification accuracy is relatively low [24]. Unlike the filter methods, the wrapper methods employ specific classifiers as the evaluation functions. Therefore, this approach is usually modeled as an optimization problem. The wrapper methods include a cuttlefish optimization algorithm [25], a new hybrid filter/wrapper algorithm for feature selection [26], a multi-objective task scheduling [27] and so on. The methods are dependent of classifiers and require the classification accuracy as the feedback to evaluate the classifiers. Therefore, the wrapper methods can achieve very high classification accuracy [25–27], but it is time-consuming. Especially, the wrapper method is applied to select features in predicting stock price direction [17]. Wrapper-based algorithms are formally transform feature selection into a single objective optimization problem [20–22]. Therefore, our motivation is to combine the advantages of two types of feature selection methods and proposes a stock prediction system that balances efficiency and performance.

In addition to high classification accuracy, past works ignored that other objectives such as optimal feature sets are also important to measure feature selection. In recent years, there are a number of effective multi-objective approaches designed for optimizing feature engineering. For example, Shone et al. introduced a hybrid model integrated deep learning and shallow learning [28]. Oliveira et al. applied NSGA for feature selection [29]. Some researchers proposed NSGA-II to build efficient feature selection models [30, 31]. Furthermore, an improved algorithm (I-NSGA-III) is utilized to select optimal feature subsets with superior performance [32].

Evolutionary algorithm is a global optimization method with high robustness and wide applicability, which has great advantages in feature selection. There have many studies explored evolutionary algorithms to transform feature selection into a single objective optimization problem [33–35] or multi-objective feature optimization problem [36–38]. Many researchers improved the performance of evolutionary algorithms by adjusting parameters or designing new solution generation methods [39–41]. Population initialization is an important part of evolutionary algorithm, which will greatly affect the convergence speed and the quality of the final solution. It is a novel research perspective to improve the performance of feature selection problems by improving the population initialization method of evolutionary algorithm [42–44]. Xue et al. [45] proposed three new initialization strategies, among which the most effective method is the hybrid initialization strategy. At the same time, the filter method is also introduced into the population initialization to improve the performance of evolutionary algorithm in solving feature selection problems [46]. Moreover, Kawamura et al. [47] proposed a method combining filter (based on correlation feature selection) and wrapper (binary particle swarm optimization) to select the optimal feature subset. However, these two methods simply use the filter method to evaluate and filter the characteristics of the original data set [46, 47].

Therefore, feature selection combined with multi-objective feature optimization was overlooked in the past works. In order to tackle the past’s weaknesses, we are motivated to propose an improved many-objective optimization algorithm integrating random forest (I-NSGA-II-RF) algorithm with a three-stage feature engineering process. The advantages of our approach are as follows. (1) Improvement strategies are explored. Our scheme offers two significant additional strategies, namely, an improved multi-objective schemes and a predefined multiple targeted search. Therefore, the algorithm proposed in this study achieves higher prediction accuracy and less computational time compare with other DL methods. (2) Feature selection is solved. There is a "black box" problem in DL methods as they cannot evaluate the importance of features [41]. An improved NSGA-II is used in our scheme for handing the many-objective feature selection that can obtain the most important features for subsequent research. (3) The multi-objectives are achieved. The prediction model has two objectives, that is, higher accuracy and lower computational complexity. Optimal feature sets are selected by feature selection engineering, which remove irrelevant and redundant features, reduce unnecessary computational overhead, and improve the predictive performance of the model.

**Fig 1:**
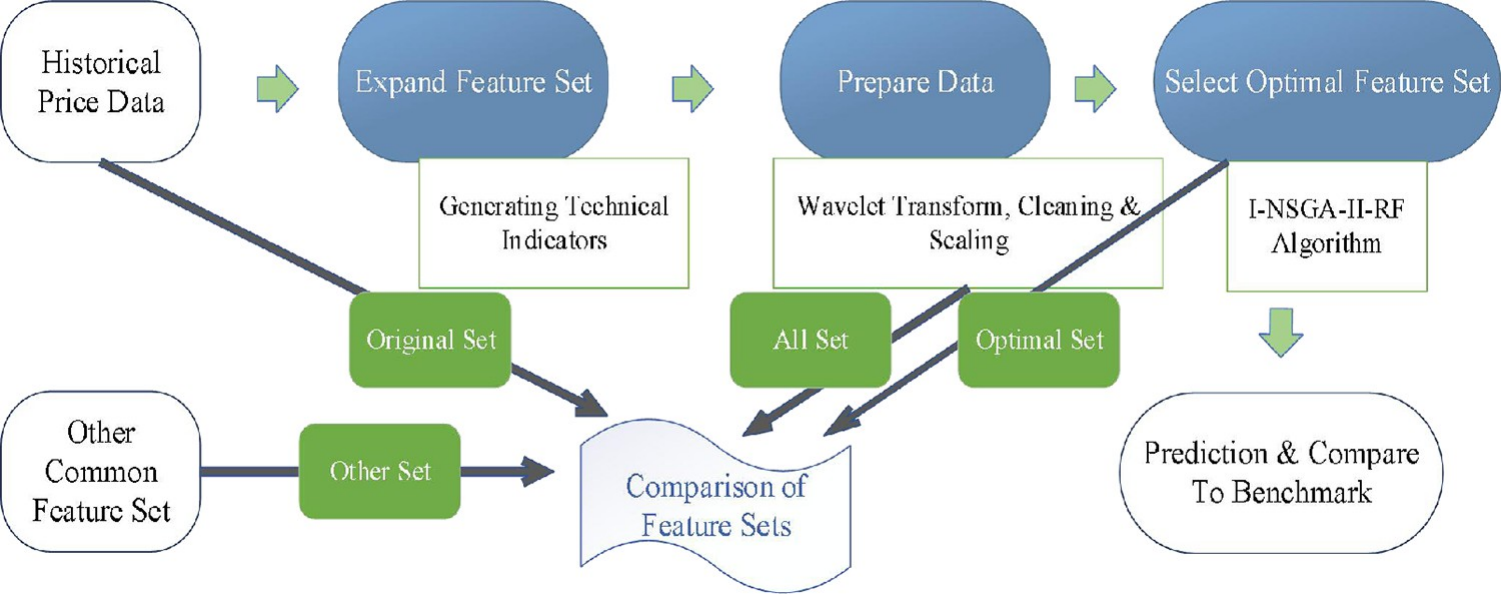
I-NSGA-II-RF stock price direction prediction system.

We propose a stock price direction prediction system applied a hybrid multi-objective evolutionary algorithm focusing on an improved feature engineering mechanism. Fig 1 shows the flowchart of the proposed I-NSGA-II-RF stock price direction prediction system. The feature engineering in this study include three stages as follows. (1) Feature set expansion by generating technical indicators. (2) Data preparation consisting of wave transform, data cleaning and normalization. (3) Optimal feature selection by applying an improved fast multi-objective genetic algorithm and random forest (I-NSGA-II-RF) algorithm. The evolutionary algorithm optimization feature selection problem can be regarded as a multi-objective problem to maximize the prediction performance and minimize the number of features. The stock price direction prediction system proposed in this study aims to reduce the amount of calculation and improve the prediction performance. In the initialization stage of NSGA-II, an improved filtering scheme integrating the filter method and the wrapper method is used to preprocess the selection of characteristic population. Then NSGA-II algorithm is used to continuously generate new solutions to adjust the structure of feature subset and random forest (classifier). Finally, the solution size and prediction accuracy of the new solution are used as evaluating indicators to select the optimal feature subset.

## 3. Methodology

### 3.1 A scheme of stock price direction prediction

Forecasting the direction of stock price change can be regarded as a binary classification problem [1]. *X*_*t*_ is a set of input characteristics on *day*_*t*_. By comparing the closing price of *day*_*t*_ and *day*_*t*+1_, *Y*_*t*+1_ can be defined as a binary value 0 or 1. The definition is expressed as Eq (1).

$$Y_{t+1}=\left\{ \begin{array}{ll} 1, & \mathrm{if}\;C_{t}<C_{t+1}\\ 0, & \mathrm{if}\;C_{t}\geq C_{t+1} \end{array}\right.$$ (1)

where *C*_*t*_ and *C*_*t*+1_ represent the closing stock price for two consecutive days. If the price of *day*_*t*+1_ is higher than the price of *day*_*t*_, it is set to up and the value is set by 1. Otherwise, it is set to down and the value is set by 0.

**Fig 2:**
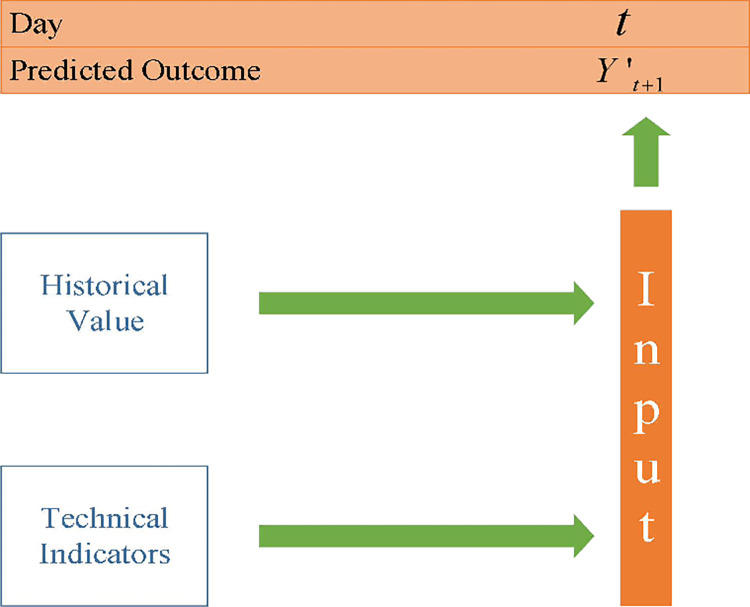
Prediction of stock price movement.

$Y_{t+1}^{\prime }$ represents the predicted direction of closing price on *day*_*t*+1_. $Y_{t+1}^{\prime }$ is a nonlinear function of the set of input features *X*_*t*_.

$$Y_{t+1}^{\prime }=f(X_{t}),$$ (2)

where *f*(·) is a nonlinear function that maps a set of input features *X*_*t*_ on day *day*_*t*_ as shown in Fig 2.

The state of input features *X*_*t*_ is affected by n previous states of prices, which can be regard as a Markov process. For *t*>*n*, the process can be expressed as:

$$p(x_{t}|x_{t-1},x_{t-2},\cdots ,x_{1})=p(x_{t}|x_{t-1},x_{t-2},\cdots ,x_{t-n}),$$ (3)

where p(A|B) represents a conditional probability of event A given event B.

Classical Markov processes assume that the past, present, and future states are independent of each other. Therefore, only the current state affects the next state in the classical Markov process as shown in Eq (4).


$$p(x_{t}|x_{t-1},x_{t-2},\cdots ,x_{1})=p(x_{t}|x_{t-1}).$$ (4)

However, external and internal factors affect the current price of the stock. The time series of stocks have the characteristics of nonlinearity and high noise. The current daily price of the stock will be affected by the status from the previous n days. So the historical data set consists of 5 input features of *day*_*t*_, that is ‘High’, ‘Low’, ‘Open’, ‘Close’, and ‘Volume’, are limited to predict the stock price direction of *day*_*t*+1_.

Therefore, the first stage of I-NSGA-II-RF stock price direction prediction system proposed in this study is to expand feature set by generating technical indicators as shown in Fig 2.

$H_{t}^{H},H_{t}^{L},H_{t}^{O},H_{t}^{C},H_{t}^{V}$ are the original historical data, which represent ‘High’, ‘Low’, ‘Open’, ‘Close’, and ‘Volume’ on *day*_*t*_, respectively. $T_{t}^{1},T_{t}^{2},\cdots ,T_{t}^{k}$ represent technical indicators which are generated as the value of nonlinear functions of the past n days’ original historical data of $H_{t}^{H},H_{t}^{L},H_{t}^{O},H_{t}^{C},H_{t}^{V}$. Then, $Y_{t+1}^{\prime }$, the predicted outcome of *day*_*t*+1_, can be attained by the original historical data and technical indicators as the result of a nonlinear function of input features of *day*_*t*_.

### 3.2 Feature set expansion by generating technical indicators

As Markovian n-states memory property mentioned in 3.1, it is reasonable to integrate historical data and technical indicators to predict the stock price direction of *day*_*t*_. Generating technical indicators to expand the input feature set to gain “the blessing of dimensionality”. High dimensional data set can be used to handle Markovian n-states to predict the stock price direction of *day*_*t*_ effectively [48].

The original 5 features of historical data include open, high, low, and close prices. The 14 common technical indicators used by researchers are as follows: Moving average convergence divergence (MACD), Commodity channel index (CCI), Average true range (ATR), Bollinger Band (BOLL), 20 day Exponential Moving Average (EMA20), 5/10 day Moving Average (MA5/MA10), 6/12 month Momentum (MTM6/MTM12), Price rate of change (ROC), Stochastic Momentum Index (SMI) and Williams’s Variable (WVAD). In addition, there are two macroeconomic technical indicators, that is, the exchange rate and interest rate. As US dollar plays the most important role in the international monetary market, US dollar index is used as the proxy for exchange rate in this study. Regarding the interest rate, the interbank offered rate in each market as the proxy is appropriate [49]. Federal funds rate in USA, Tokyo Interbank Offered Rate (TIBOR), Hong Kong Interbank Offered Rate (HIBOR), Shanghai Interbank Offered Rate (SHIBOR) and Mumbai Interbank Offered Rate (MIBOR) are used as technical indicators. The details are shown in Table 1.

**Table 1:** Feature sets.

types	Feature sets
Historical data	open, high, low, and close prices
Technical indicators	Moving average convergence divergence (MACD), Commodity channel index (CCI), Average true range (ATR), Bollinger Band (BOLL), 20 day Exponential Moving Average (EMA20), 5/10 day Moving Average (MA5/MA10), 6/12 month Momentum (MTM6/MTM12), Price rate of change (ROC), Stochastic Momentum Index (SMI) and Williams’s Variable (WVAD).
Macroeconomic indicators	exchange rate and interest rate

There are 81 technical indicators generated out of the original 5 input features to get the expanded feature set, that is, TA-Lib. In recent years, TA-Lib is popular among market traders and researchers to perform technical analysis of financial time series [17]. Therefore, all technical indicators in this study are computed using the functions provided by TA-Lib library (TA-Lib, 202)). All generated technical indicators can be classified as one of 6 function groups of indicators. They are Overlap Studies, Momentum Indicators, Volume Indicators, Volatility Indicators, Price Transform, and Cycle Indicators [17].

### 3.3 Data preparation: Wavelet transform, data cleaning and normalization

Stock price time series is high-noise, nonlinear and non-stationary. Data preparation is a key stage of feature engineering. In recent studies, some researchers applied WT to extract multi-frequency features of time series as data preparation to forecast stock index [50, 51]. Wavelet transform (WT) can effectively decomposed historical data into different frequency segmentation. Each frequency band does not overlap one another. The decomposed frequency range includes all frequency bands of the original time series. Historical stock price data have continuous variables with different measurement units for volume and closing prices. Furthermore, some technical indicators are rate measurements. Data normalization is a crucial step that transform all input data into a homogenous numerical array. The new array will be fed into the I-NSGA-II-RF algorithm. Eq (5) is used to process different scaled features. As data normalization precisely preserves all relationships in the data, and thereby, it avoids any bias [52].


$$x_{norm}=\frac{x-x_{min}}{x_{max}-x_{min}}.$$ (5)

### 3.4 Optimal feature selection by the I-NSGA-II-RF algorithm

The original historical data and the generated technical indicators constitute a total of 81-dimensional data which are used as input features in this study. However, the high-dimensional data may affect the classification speed and even lower the classification accuracy. It is necessary obtain optimal low-dimensional feature subsets in high-dimensional space. Feature selection and feature extraction are the two commonly used methods to find a suitable feature subset. Feature extraction will transform the existing features into new features. The transformation process will lose the interpretability and understandability of the original data. Comparatively, feature selection only chooses the most suitable feature subset.

A stock price direction prediction system considering both efficiency and performance is proposed. I-NSGA-II-RF algorithm combine the advantages of filter and wrapped methods. I-NSGA-II-RF algorithm is wrapped-based method, which improves the performance of the classifier by removing irrelevant redundant features. In the initialization phase of I-NSGA-II, two filtering methods are integrated to preprocess the selection of characteristic population, which further improves the efficiency of the algorithm.

#### 3.4.1 Random forest algorithm

Random forest is an integrated learning method. Results of RF are generated by many decision tree votes. Multiple trees are suitable for parallel computing. The computing speed is fast, and it is not easy to generate over fitting problems. Therefore, they are more suitable for large-scale classification problems [52]. Its basic training process is to extract multiple samples from the data set by means of put back sampling, build a decision tree for each sample, and finally get the prediction results by means of collective voting of all decision trees. A group of decision trees (*h*_1_(*x*), *h*_*z*_(*x*),⋯,*h*_*n*_(*x*)) consist of a random forest. The margin function of RF is expressed as follows:

$$\mathrm{marg}\left(X,Y\right)={\mathrm{avg}}_{n}\left(I(h_{n}(X)=Y)\right)-\underset{j\neq Y}{max}\;{\mathrm{avg}}_{n}\left(I(h_{n}(X)=j)\right),$$ (6)

where marg (·) represents margin function. *I*(·) represents indicator function, avg (·) represents averaging function, ***j*** represents wrong category vector, *X* represents input vector, *Y* represents correct category vector and *n* represents number of trees. Eq (8) indicates that the maximum average number of votes that classify the input variable *X* as the correct category vector (*Y*) exceeds the category vector (***j***) that is classified as the wrong. According to the theorem of large numbers, when the number of trees increases to a certain extent, the generalization error E* will be less than a fixed value. The equation is as follows:

$$E^{*}=P_{X,Y}(marg(X,Y)<0).$$ (7)

Since RF adopts repeated sampling with return, about 63% of the data is repeatedly sampled. The other 37% of the last extracted data is called out of bag data. RF uses out of bag (OOB) to evaluate generalization ability, and the resulting error is the out of bag error *B*_error_.

#### 3.4.2 I-NSGA-II based on filtering and external archiving

Many data indicators are input into the RF algorithm. This may cause the problem of high indicator correlation and the difficulty of distinguishing the of indicators’ importance, resulting in the performance degradation of the classifier. I-NSGA-II is used to improve RF. The improvement methods include deleting irrelevant and redundant indicators and optimizing the combination of technical indicators. In addition, the improvement methods also include improving the population initialization method of NSGA-II and adding an external archiving mechanism. These improvement strategies can maintain the diversity of solutions and improve the performance of multi-objective evolutionary algorithm. The strategies also synchronously optimize the key parameters of the classifier to achieve the effect of synchronous optimization, which can improve the prediction performance of the classifier.

#### 3.4.3 Fitness valuation

The process of multi-objective optimization is to find the decision vector X* and make it have the optimal fitness.


$$X^{*}=(x_{1}^{*},x_{2}^{*},\cdots ,x_{D}^{*}),$$ (8)

Another objective is to minimize the function vector.


$$min\;F(X)=(f_{1}(X),f_{2}(X),\cdots ,f_{m}(X)),$$ (9)

The constraints of the decision vector are as follows:

$$\left\{ \begin{array}{ll} g_{i}(X)\geq 0, & i=1,2,\cdots ,Q\\ h_{j}(X)=0. & j=1,2,\cdots ,P \end{array}\right.$$ (10)

Different from the single objective problem, the multi-objective problem can not find an X in the decision space, which is optimal for all the objective fitness. The reason is that the constraints are often in conflict with each other. In terms of multi-objective feature selection, the optimal decision vector represents the solution with higher classification accuracy and fewer features. Therefore, the number of selected features and the accuracy of classification are the two objectives of the algorithm proposed in this study.

The objective function for selecting the number of features is expressed as follows:

$$f_{1}(Z)=\frac{1}{D}\sum_{j=1}^{D}\;z_{j}.$$ (11)

Eq (12) is the calculation formula of classification accuracy:

$$f_{2}(Z)=(\frac{1}{l}\sum_{l=1}^{n}\;\frac{N_{Car}}{N_{All}})\times 100\%,$$ (12)

where *Z* is the decoding scheme, D is the dimension (number of features), *N*_*Car*_ is the number of correctly predicted samples, and *N*_*All*_ is the number of all samples.

### 3.4 A hybrid algorithm of I-NSGA-II-RF

#### 3.4.1 Hybrid initialization

The I-NSGA-II algorithm proposed in this study enables feature selection and classifier structure optimization synchronous. The improved strategies accelerate the convergence speed of the algorithm, shorten the running time, and enhance the performance of the algorithm.

**Fig 3:**
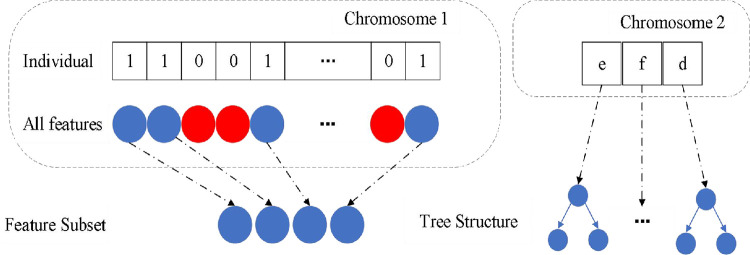
Individual representation.

As shown in Fig 3, the method of multi-chromosome mixed coding is adopted. The first chromosome encodes all features. The length of the chromosome is equal to the number of features in the data. Value 0 means that the feature is not selected, and value 1 means that the feature selected will be retained. The second chromosome encodes the key parameters of the random forest classifier. These parameters in Fig 3 include: e represents the number of trees in the random forest, f represents the maximum characteristic number, and d represents the maximum depth. Then, according to the selected feature subset and classifier parameters, the corresponding random forest model is trained. The selected feature number and accuracy will be used to evaluate the optimization and continue to produce better solution sets. These processes will promote the individuals in the population to find the global optimization.

Filter method is independent of the classifier and uses some specific evaluation criteria to evaluate the features. Statistical method, such as Chi-square test, can perform correlation analysis on classification variables [20–22]. Therefore, Chi-square is adopted in the initialization phase of NSGA-II algorithm. Chi-square is used to evaluate features first, then extract features more relevant to the target from all feature variables and reduce redundant features to initialize the population to improve performance [53]. The equation is as follows:

$$x^{2}=\frac{{\left(f_{0}-f_{e}\right)}^{2}}{f_{e}},$$ (13)

where *o* stands for observation, that is, the actual frequency. *e* stands for expectation, that is, the expected frequency of X assuming that the variable x is independent of the target variable y. The theoretical value refers to the theoretical frequency. First, the Chi-square value of each characteristic variable to the target variable is obtained by evaluating the characteristics through the Chi-square equation. Then, the Chi-square value is sorted, and the top ranked features are selected. These selected features have higher correlation with the target variable y [54].

During the initialization of Chi-square algorithm, most of the features with high scores and a small part of irrelevant features will be retained. These two choices are to maintain the diversity of initialization from the perspective of the interaction between features. Basically, 80% of the most useful features are selected through experimental comparison. Therefore, how to get the appropriate features from the selected features and determine the appropriate number of features are the key points to be considered.

Hybrid initialization is used to solve this problem [45]. Most individuals are initialized with the first 80% of the selected features, and the rest are initialized with the last 20% of the features. The processes of hybrid initialization are as follows. First, Chi-square values are sorted. Then the 80% of the features with the highest Chi-square value from the initial features will be selected and saved in WR. As for the 80% of all individuals, the features will be retained if the features selected by the individual are saved in WR as well as in the initial matrix (the corresponding value is 1). As for the 20% of all individuals, the features will be retained if the features selected by the individual are not WR but the features are selected by the initial matrix.

Algorithm 1: hybrid initialization

    Population size *ps*, characteristic number *D*, initial matrix *XG*, first 80% characteristic matrix *WR*, record matrix *PF*.

1.*for i*≤*ps do*

2.        *for* 80% *individuals do*

3.            *for j*≤*D do*

4.                *if* (XG_*ij*_ = = 1) *and* (*j*∈*WR*) *then*

5.                    *PF*_*ij*_ = 1;

6.            *else*

7.                *PF*_*ij*_ = 0;

8.            *end*

9.        *end*

10.        *for* 20% *individuals do*

11.            *for j*≤*D do*

12.                *if* (XG_*ij*_ = = 0) *and* (*j*∈*WR*) *then*

13.                    *PF*_*ij*_ = 1;

14.                *else*

15.                    *PF*_*ij*_ =0;

16.            *end*

17.        *end*

18.    *end*

#### 3.4.2 Integrated learning population initialization

In order to improve the ability of filter method, XGBoost is applied as a pre training model in the process of population initialization. XGBoost stands for extreme gradient boosting. As an embedded method, the algorithm has good performance high processing speed by exploiting all available hardware resources [55].

The importance score of each feature can be obtained directly based on XGBoost, which measures the importance of the feature in a tree structure. The score is derived for a wider range of objective functions. It is similar to the impurity score for evaluating decision trees.

In regard to a single decision tree, the feature importance of each tree is calculated according to the quality that the feature improves the performance. As for the tree ensemble of XGBoost, the greater the performance improvement of a single feature, the greater the weight, and will be selected by more lifting trees, the higher the importance. Comparatively, as for the tree ensemble of XGBoost, the greater the degree of performance improvement of features, the greater the weight assigned to them. These features will be selected by more trees, and the more important they will be. Finally, according to the importance of features in all trees, the weighted sum and average value are obtained to obtain the final importance score. Since XGBoost and Chi-square adopt different evaluation methods, they will get different evaluation results. The information obtained by integrating the two parts of heuristic information is more applicable. The initialization process of integrated learning population by combine XGBoost and Chi-square is shown in Algorithm 2 as follows.

Algorithm 2: integrated learning population initialization.

1. The chi-square value of the feature is evaluated by the Chi-square method. The features are arranged from large to small according to the chi square value.

2. The initial population a is obtained by algorithm 1.

3. Population 1 is used as the input of random forest. The TPE algorithm [56] is used to optimize the parameters for 50 times.Then the new population a’ with the original population and accuracy is obtained.

4. The importance of features is evaluated by XGBoost method to get corresponding scores. The features are ranked from large to small according to the score of importance.

5. The initial population b is obtained by algorithm 1.

6. Population 2 is the input of random forest. The TPE algorithm is used to optimize the parameters for 50 times. Then the new population b’ with the original population and accuracy is obtained.

7. The accuracy in a’ and b’ is sorted. The first 50% individuals without accuracy were selected as the final initial population P.

#### 3.4.3 External archiving mechanism

The external archiving mechanism is used to divide the population into two populations, that is, the working population and the external archiving guidance population. The algorithm performs three rounds of screening. First, the first round of screening is conducted according to the dominance relationship to remove the solutions with low fitness. And the rest are added to the archive. Secondly, the second round of filtering is carried out according to the dominance relationship in the archive to further remove the solutions with low fitness. Then the position of archived individuals in the grid is calculated. Finally, if the number of archives exceeds the archive threshold, the solutions are filtered according to the congestion distance. Consequently, the solution information is updated in the archive. The specific steps are shown in Algorithm 3.

Algorithm 3: external archiving mechanism.

1. for the new solution x, if archive is empty, then x is saved in archive. Otherwise, go to the next step;

2. if x is dominated by all solutions in the archive, the archive remains unchanged. Otherwise, go to the next step;

3. if x dominates the decomposition in the middle of the archive, delete the dominated solution and do not save it. Otherwise, go to the next step;

4.If x and all solutions in the archive do not control each other, save x to judge whether the archive size reaches the maximum value. If not, the update ends. Otherwise, calculate the congestion distance of each solution in the archive, delete the solution with the minimum congestion distance, and the update ends.

#### 3.4.4 I-NSGA-II-RF algorithm

**Fig 4:**
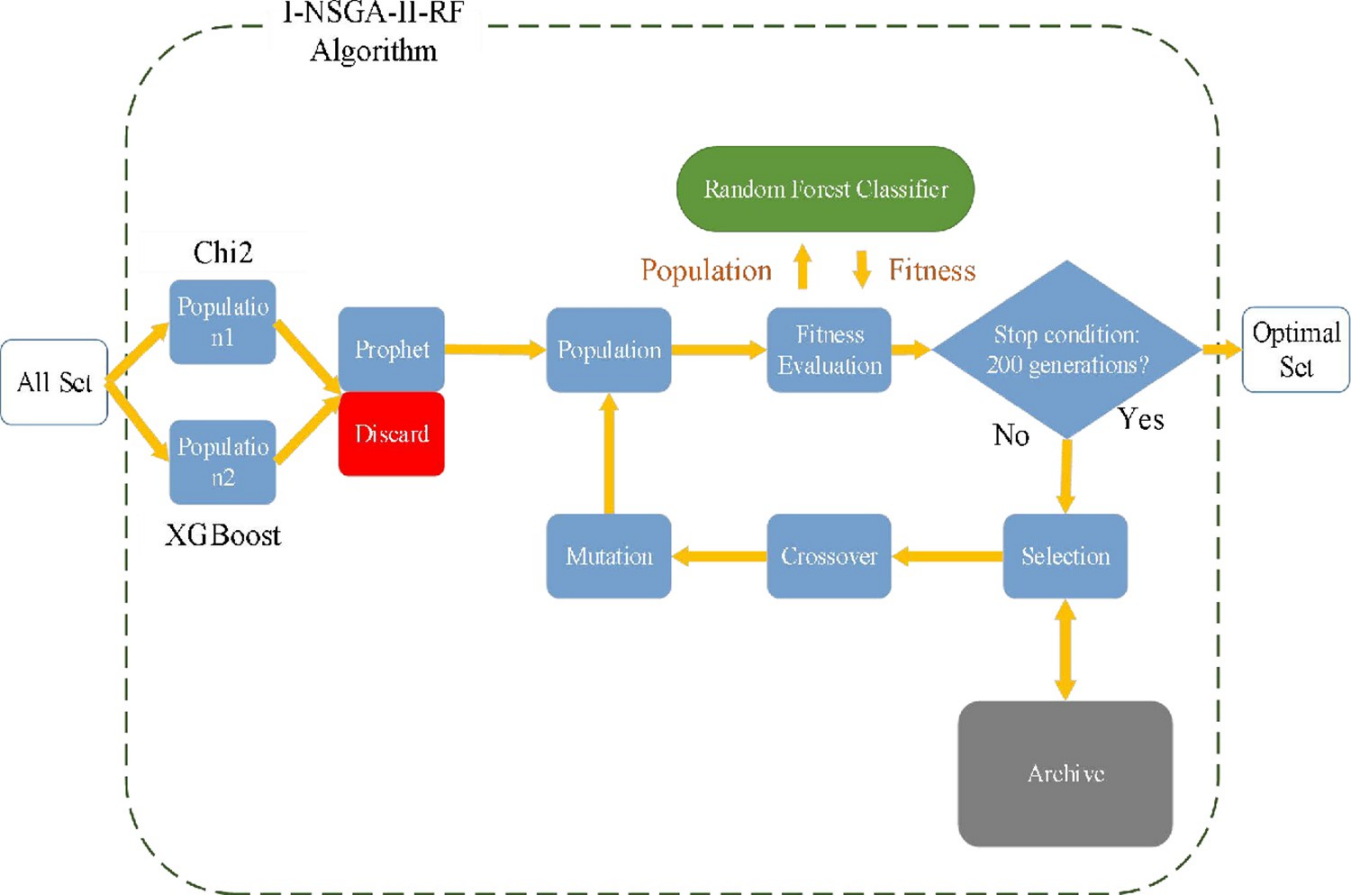
I-NSGA-II-RF algorithm.

The operation process of I-NSGA-II-RF algorithm shown in Fig 4 is illustrated as follows. According to the solution set information with the highest accuracy in each generation, the optimal search approximate optimal or optimal solution is collected and updated. Subsets are used as inputs to the classifier to minimize the number of selected features while maximizing accuracy.

The initial population has 20 chromosomes. Each chromosome has two chromosomes that control the three key parameters of the random forest structure. The two functions of the multi-objective algorithm are the accuracy of all individuals in a generation of population evaluated by RF training and the number of individuals selected by the current individual. Finally, the optimal solution is found through crossover and mutation.

## 4. Experimental results and discussion

### 4.1. Experimental environment and data description

**Fig 5:**
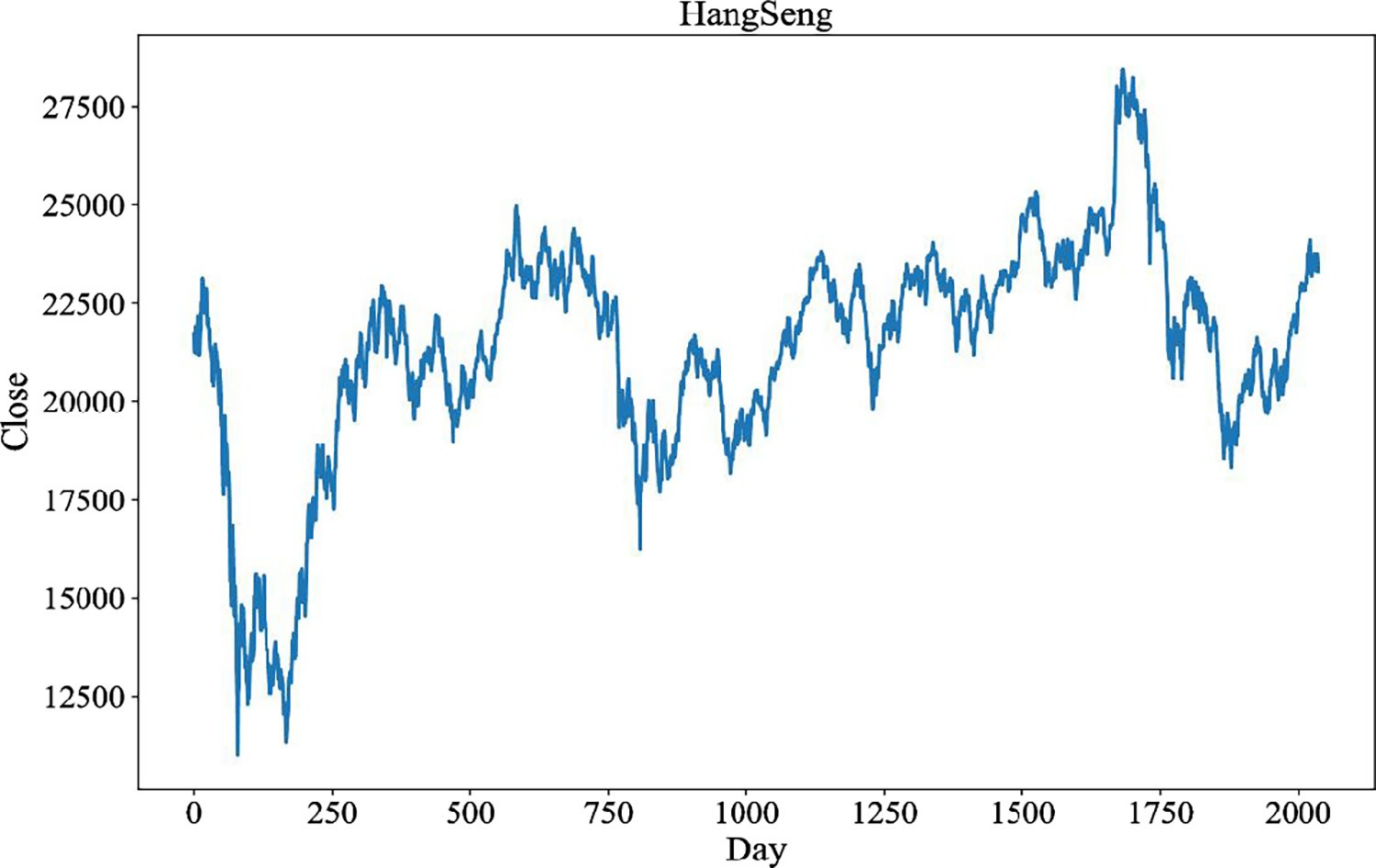
Historical closing price of Hang Seng Index.

All the experiments were performed on the following computers. Hardware information are as follows: Intel i5-9500, 3.00GHZ processor and 8GB RAM. Software information are as follows: Python 3.8.5, Visual Studio Code 1.67.1 and Jupyter notebook 6.4.6. The six stock indices that is, DJIA, S&P500, Hang Seng, Nikkei 225, CSI 300, and Nifty50, are chosen as datasets to predict the stock index’s up or down movement direction on the next day. All the experiments were performed on the following computers. Hardware information are as follows: Intel i5-9500, 3.00GHZ processor and 8GB RAM. Software information are as follows: Python 3.8.5, Visual Studio Code 1.67.1 and Jupyter notebook 6.4.6. Five historical data, including High Price (High), Low Price (Low), Opening Price (Open), Closing Price (Close), and Trading Volume (Volume), were provided by a global financial portal: Investing.com. The time period of the datasets are from 6/1/2008 to 9/30/2016, which is 2038 trading days. Table 2 shows the statistical information of the Hang Seng dataset. Fig 5 shows the historical closing price of the Hang Seng Index during this period.

**Table 2:** Description of Hangseng dataset.

	Open	High	Low	Close	Volume
count	2038	2038	2038	2038	2038
mean	21365.14021	21494.34245	21206.40291	21350.90627	177860.7163
Std	2821.602357	2799.315553	2839.360869	2814.073328	65544.77044
Min	11624.37333	11994.72047	11133.13161	11678.27805	-18108.78173
25%	20189.8654	20307.57736	20046.93148	20175.07828	142071.4852
50%	21783.65234	21904.63445	21647.66647	21780.34749	165589.252
75%	23138.20986	23249.39016	23024.22981	23128.13668	198446.579
Max	28225.01586	28312.28772	28077.5651	28271.48134	871670.5834

### 4.2 Feature set expansion by generating technical indicators

Two expanded feature sets are created From the original 5 features of historical prices by generating technical indicators as described in Section 3.2. The expanded feature set 1 adds 16 common technical indicators. The expanded feature set 2 adds 67 technical indicators created from the original input features to capture the blessing of dimensionality. As described in Section 3, original historical values represent the current day’s prices Xt while technical indicators retrospect the past 14 days price information from day t in this study. Therefore, a predicted result of day t +1 is a nonlinear function of input features of day t, which includes the historical prices of day t and the technical indicators of the period of days t-14, t-13,⋯, t-1, t.

### 4.3 Wavelet transform, data cleaning and normalization

**Fig 6:**
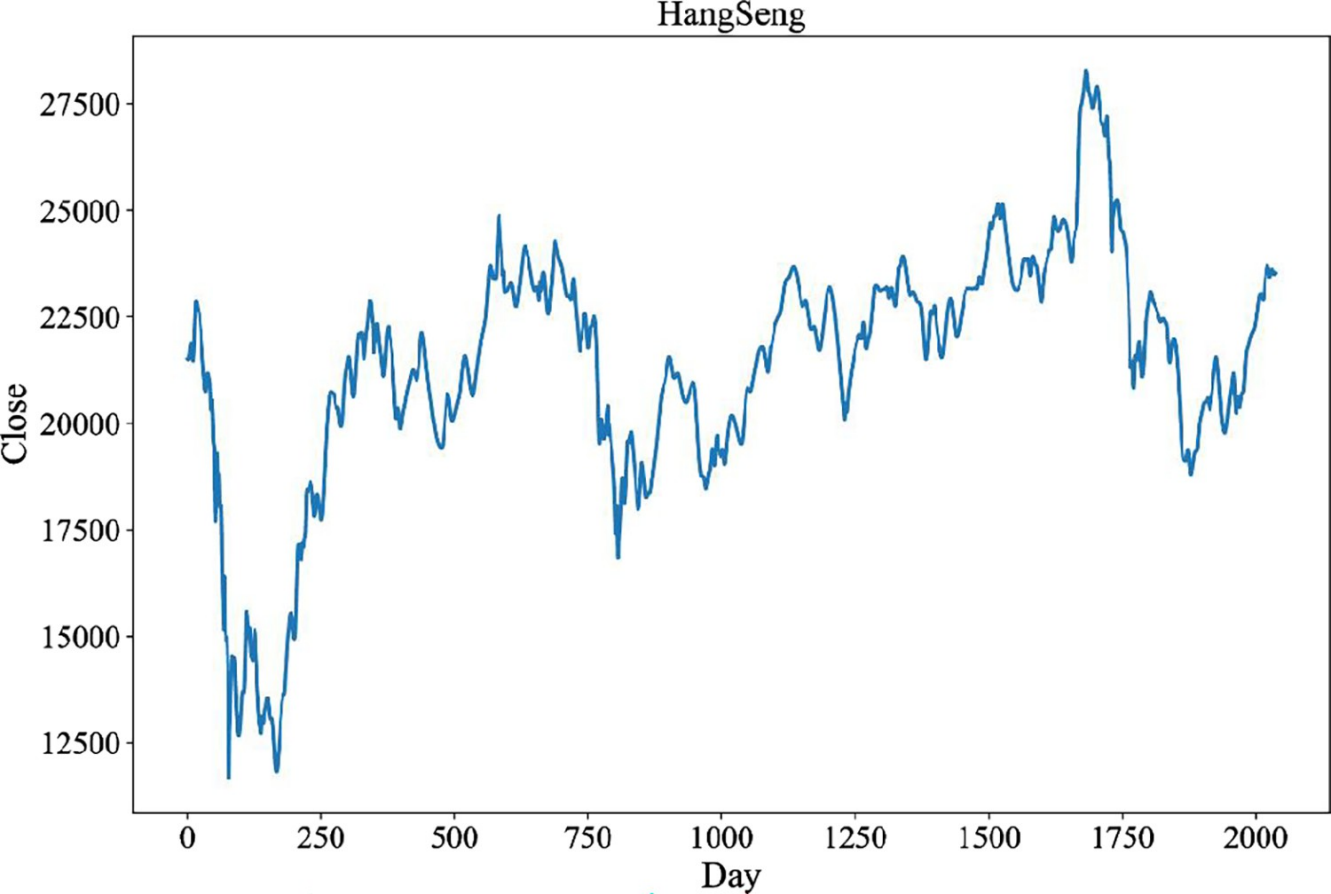
The noise-reduced closing price of Hang Seng Index.

Compared with Fourier transform, wavelet transform (WT) can simultaneously analyze the frequency components of financial time series Therefore, WT can effectively deal with unstable financial time series. The pywt library is used to decompose the index price sequence into time domain and frequency domain. The noise-reduced sequence is shown in Fig 6.

A new column “trend” is added in the expanded sequence. The output of the model is the movement direction of the stock price from the past day to the current day. So “trend” is a binary feature consisting of 0 and 1. The first 88 null values caused by the expansion feature are deleted from 2038 trading days. Then the remaining 1950 trading days are used as the data set. All data sets are divided into training sets and test sets, in which training sets account for 80% (the first 1560 trading days) and test sets account for 20% (the next 390 trading days). The test set is used to evaluate the final selected feature subset. The training set is divided into the first 85% (1326 trading days) training model and the last 15% (234 trading days) to verify the model. All values are mapped between [0,1] according to Eq (5).

### 4.4 Parameter setting

The parameter settings used in this experiment are shown in Table 3.

**Table 3:** Parameter settings.

parameter type	parameter settings
evolutionary algebra	200
population size	20
Individual chromosome number	2
external archive	200
crossover rate	1
Variation rate	1/n (n is the population size)

### 4.5 Evaluation function

The following common classification indicators are adopted in this study to measure the performance of the classifier. TP is the true rate. TN is the true negative rate. FP is the false positive rate. FN is the false negative rate.


$$Accuracy=\frac{TP+TN}{TP+TN+FP+FN},$$ (14)

$$Precision=\frac{TP}{TP+FP},$$ (15)

$$Recall=\frac{TP}{TP+FN},$$ (16)

$$F1=2\times \frac{\mathrm{Precision}\times \mathrm{Recall}}{\mathrm{Precision}+\mathrm{Recall}},$$ (17)

$$AUC=\int_{0}^{1}\;\frac{TP}{TP+FN}d\frac{FP}{FP+TN}.$$ (18)

### 4.6 Comparison of different classifiers

**Fig 7:**
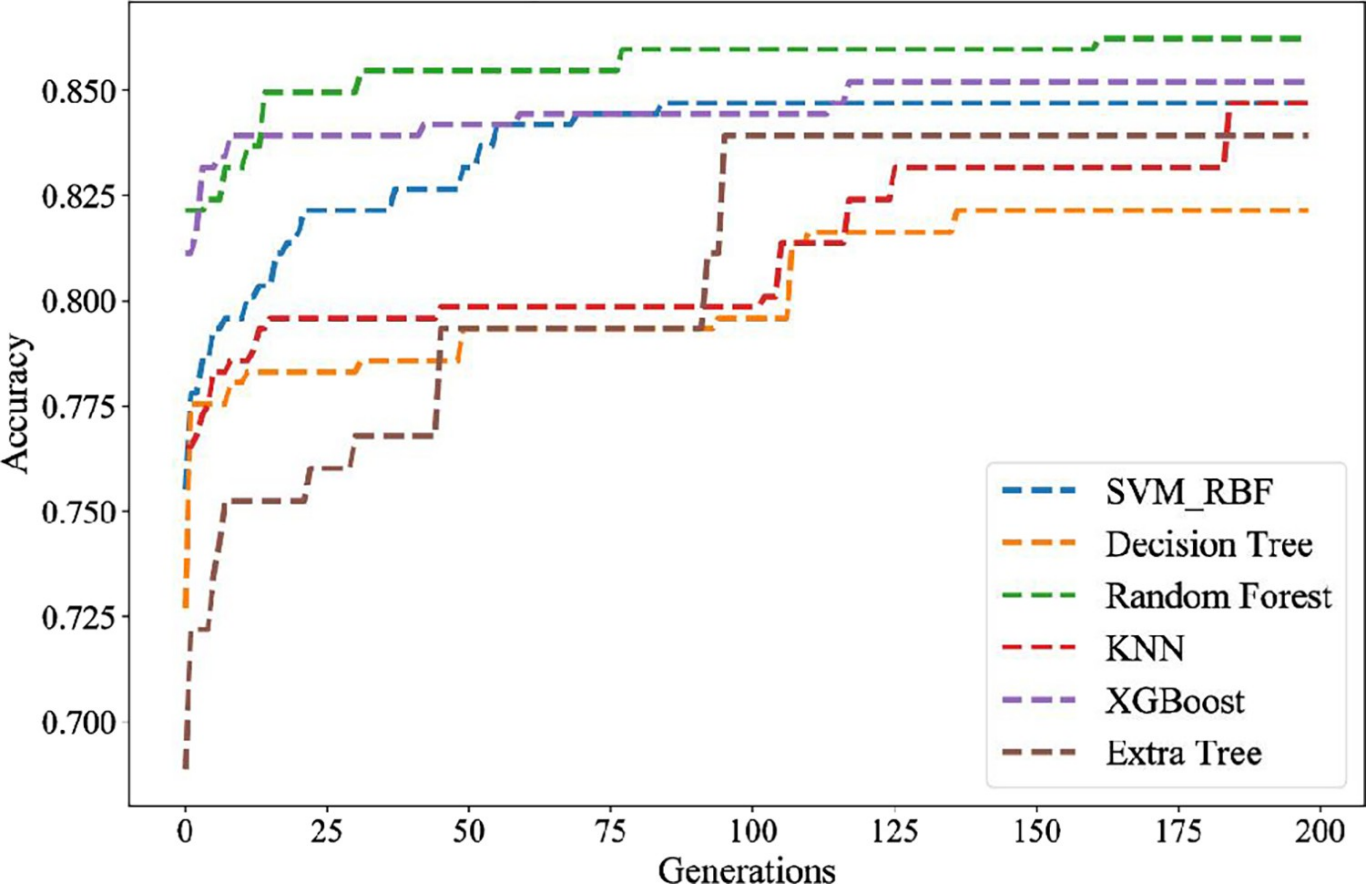
Comparison of different classifiers of s&p500.

In order to compare the performance of the I-NSGA-II-RF prediction model, the 200 generations of each machine learning model are optimized by using the I-NSGA-II proposed in this paper. The limit tree, XGBoost, KNN, decision tree, support vector machine with RBF kernel and random forest model compared in the experiment. The first 85% (1326 trading days) of the training set is used as the training set, and the last 15% (234 trading days) is used as the verification set. The verification accuracy of RF model is 86.54%, which is the highest among the ML models. Therefore, the advantage of hybrid I-NSGA-II-RF algorithm is promising. The experimental results of s&p500 index are shown in Table 4 and Fig 7.

**Table 4:** Comparison of different classifiers of s&p500.

Model	Accuracy (%)
Random Forest	**86.54**
Decision Tree	82.14
Extra Tree	83.93
KNN	84.69
XGBoost	85.20
SVM_RBF	84.69

### 4.7 Comparison of different feature subsets

Hang Seng Index was used to compare the characteristics of subsets. We test the daily data added with a total of 5 feature datasets (original datasets), all 81 feature datasets (all feature datasets) generated through feature expansion, 18 feature datasets commonly used in other studies (other datasets) [57], and 2 feature datasets (the best subset datasets) selected by I-NSGA-II-RF proposed in this study. The results are shown in Table 5. All feature sets are used as input variables to train through random forest model. The accuracy was tested after 20% (234 trading days) of the retained dataset. The prediction accuracy of the original historical data set is 76.96%. The accuracy of the feature set commonly used in other studies reaches 82.17%. The accuracy of the 81 expanded data sets is only 57.83%, which is the worst. This shows that too many data set features may affect each other. Then a large number of redundant features will affect the performance of the classifier. The feature subset selected by I-NSGA-II-RF has only two features, with an accuracy of 89.44%, an F1 score of 90.06% and an AUC of 89.4. All indicators are better than other feature subsets. The experimental results show that I-NSGA-II-RF effectively reduces irrelevant features and redundant features. The performance of the classifier is greatly improved, and the prediction effect is the best by using the selected optimal subset.

**Table 5:** Comparison of different feature subsets of Hang Seng data set.

Feature Sets	Num of Input features	Accuracy (%)	F1- score (%)	AUC
Original Set	5	76.96	80.59	75.83
ALL Set	81	57.83	69.01	54.66
Other Set	18	82.17	81.78	82.17
Optimal Set	**2**	**89.44**	**90.06**	**89.4**

### 4.8 Comparison of single objective, multi-objective and improved multi-objective

#### 4.8.1 Comparison of classification indicators

**Fig 8:**
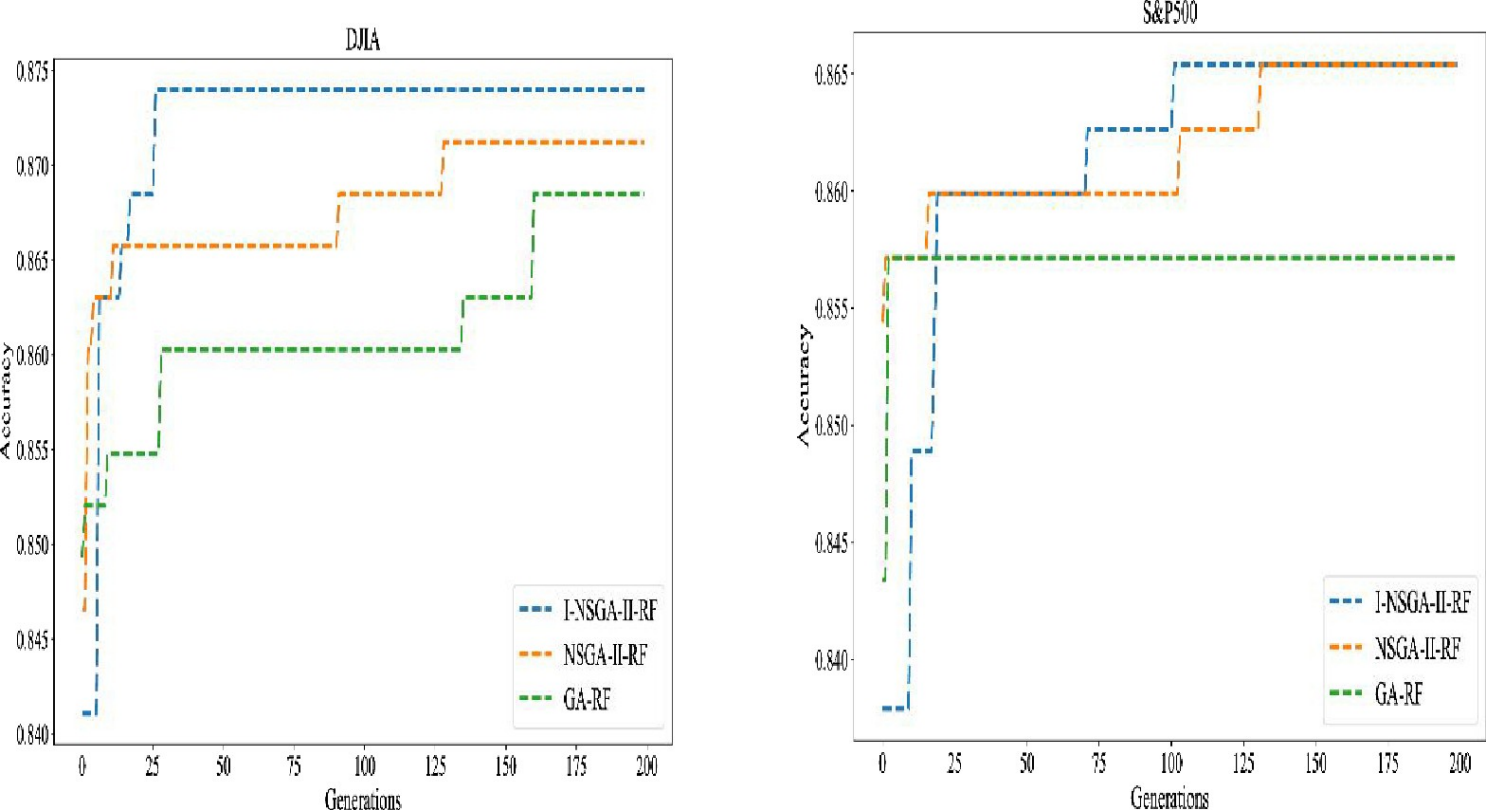
Evolution process of different feature selection algorithms of DJIA and s&p500.

**Fig 9:**
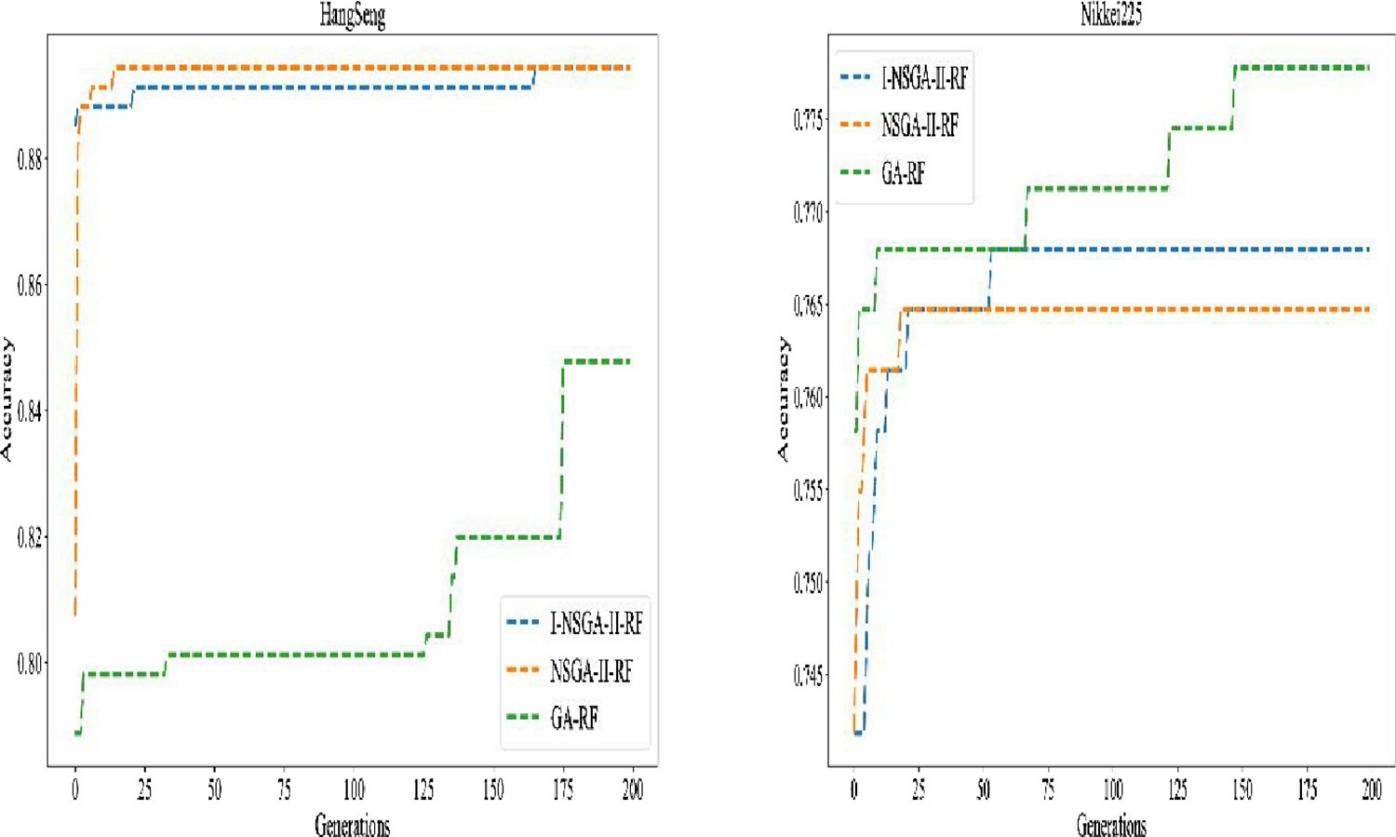
Evolution process of different feature selection algorithms for Hangseng and Nikkei225.

**Fig 10:**
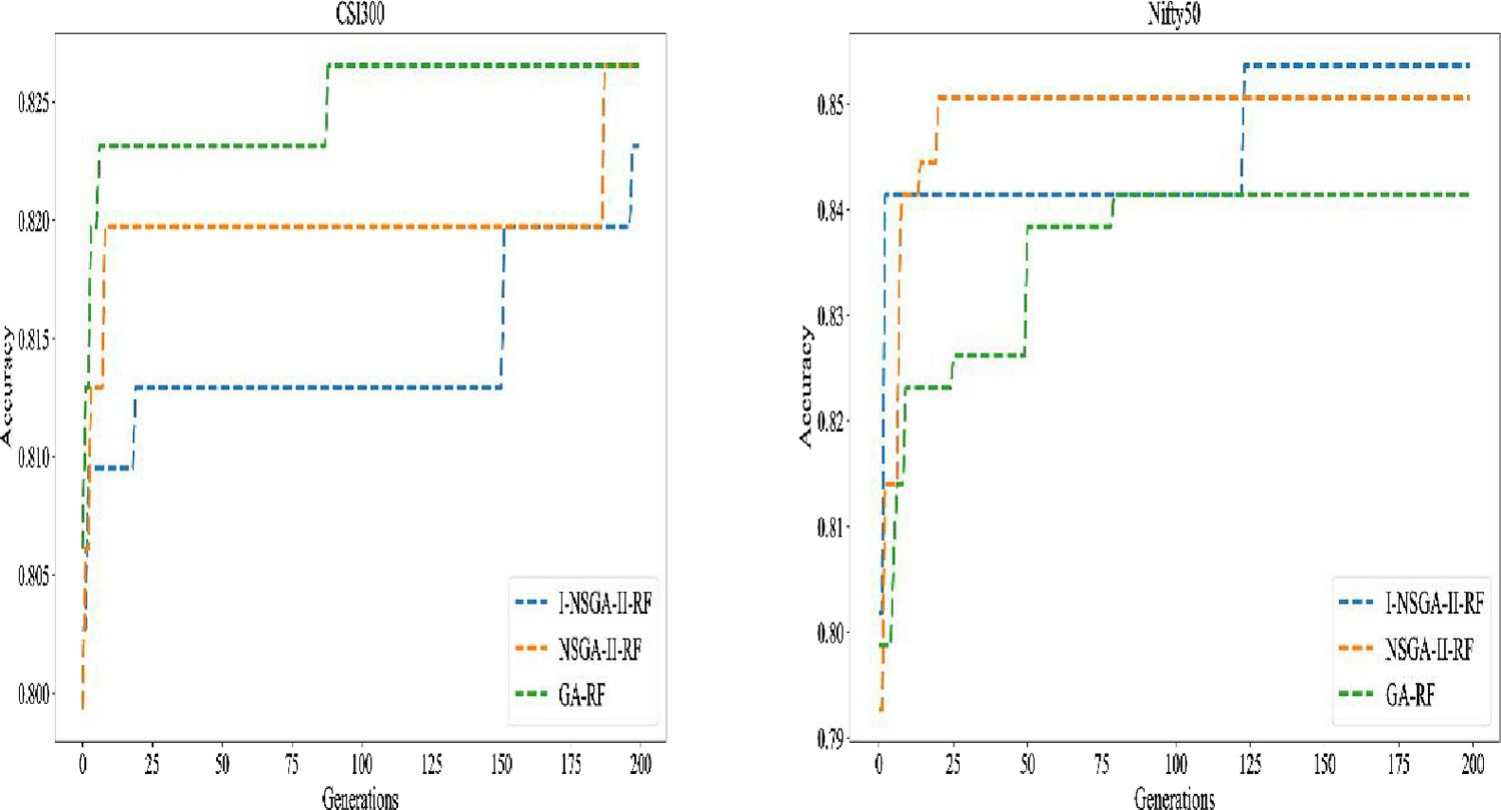
Evolution process of different feature selection algorithms of CSI300 and Nifty50.

The single objective-based method (GA), the multi-objective based method (NSGA-II) and the improved multi-objective based method (I-NSGA-II) are used to test six data sets. The experimental results are shown in Table 6. It can be seen from the table that I-NSGA-II and NSGA-II achieved the same results in s&p500 and Hang seng index data sets. NSGA-II and GA algorithms achieve the same results in CSI300 index data set. I-NSGA-II algorithm obtains 4, 3 and 4 best in accuracy, F1 score and AUC among six stock price index data sets respectively. NSGA-II algorithm obtains 4, 4 and 4 best in accuracy, F1 score and AUC among six stock price index data sets respectively. The specific numerical difference is very small. It can be considered that the two algorithms are equal in performance. The GA algorithm achieves 2, 1 and 2 optimal results respectively. The experimental results show that the multi-objective algorithm has better performance than the single objective algorithm. From the number of features selected, the average number of features selected by multi-objective algorithm (I-NSGA-II and NSGA-II) is 14.5, while the average number of features selected by single objective algorithm (GA) is 32.5. Therefore, the multi-objective algorithm greatly reduces the number of selected features, which improves the ability of feature selection process, and improves the performance of the classifier. The changes of fitness verification accuracy during the training of the three optimization algorithms are shown in Fig 8, Fig 9, Fig 10.

**Table 6:** Comparison of single objective, multi-objective and improved multi-objective.

**I-NSGA-II**	Num of features	Accuracy (%)	F1-score (%)	AUC
DJIA	17	**87.4**	**88.27**	**87.28**
S&P500	4	**86.54**	**87.9**	**86.3**
HangSeng	2	**89.44**	**90.06**	**89.4**
Nikkei225	40	76.8	76.72	76.84
CSI300	6	82.31	82.67	82.33
Nifty50	6	**85.37**	87.5	**85.09**
**NSGA-II**	Num of features	Accuracy (%)	F1-score (%)	AUC
DJIA	3	87.12	87.92	87.05
S&P500	6	**86.54**	**87.9**	**86.3**
HangSeng	23	**89.44**	**90.06**	**89.4**
Nikkei225	33	76.47	76.16	**76.49**
CSI300	4	**82.65**	**83.17**	**82.68**
Nifty50	30	**85.06**	**87.53**	84.26
**GA**	Num of features	Accuracy (%)	F1-score (%)	AUC
DJIA	32	86.85	88.06	86.55
S&P500	46	85.71	87.79	84.86
HangSeng	16	84.78	86.35	84.39
Nikkei225	39	**77.78**	**77.33**	77.78
CSI300	32	**82.65**	82.11	**82.64**
Nifty50	30	84.15	86.87	83.14

#### 4.8.2 Comparison of running time

As can be seen from Table 7, the I-NSGA-II algorithm proposed in this study requires the least running time. Compared with the NSGA-II algorithm, the average running time is shortened by 70.85%. Furthermore, compared with the single objective GA algorithm, the average running time is shortened by 87.66%. The comparisons indicate that the computing performance of the data set optimized by I-NSGA-II algorithm is greatly improved. Moreover, the running time variance of I-NSGA-II algorithm is smaller. The variance difference between NSGA-II algorithm and GA algorithm is rather small. The stability of I-NSGA-II algorithm in running time further proves that it performs stably and effectively under different market conditions. Although compared with other algorithms, I-NSGA-II integrates the information of two filtering feature selection in population initialization to introduce a priori population, it spent less time. The improvements come from the appropriate random forest tree structure and the selection of fewer features. These two advantages avoid a large amount of unnecessary computing overhead and enhance the operation efficiency.

**Table 7:** Comparison of running time.

	Max	min	average	sd
I-NSGA-II	296.306293	204.7432599	253.5435231	38.67589313
NSGA-II	1292.459256	289.733876	868.3281864	333.9695651
GA	2379.499885	1492.98367	2051.890502	331.9404655

**Fig 11:**
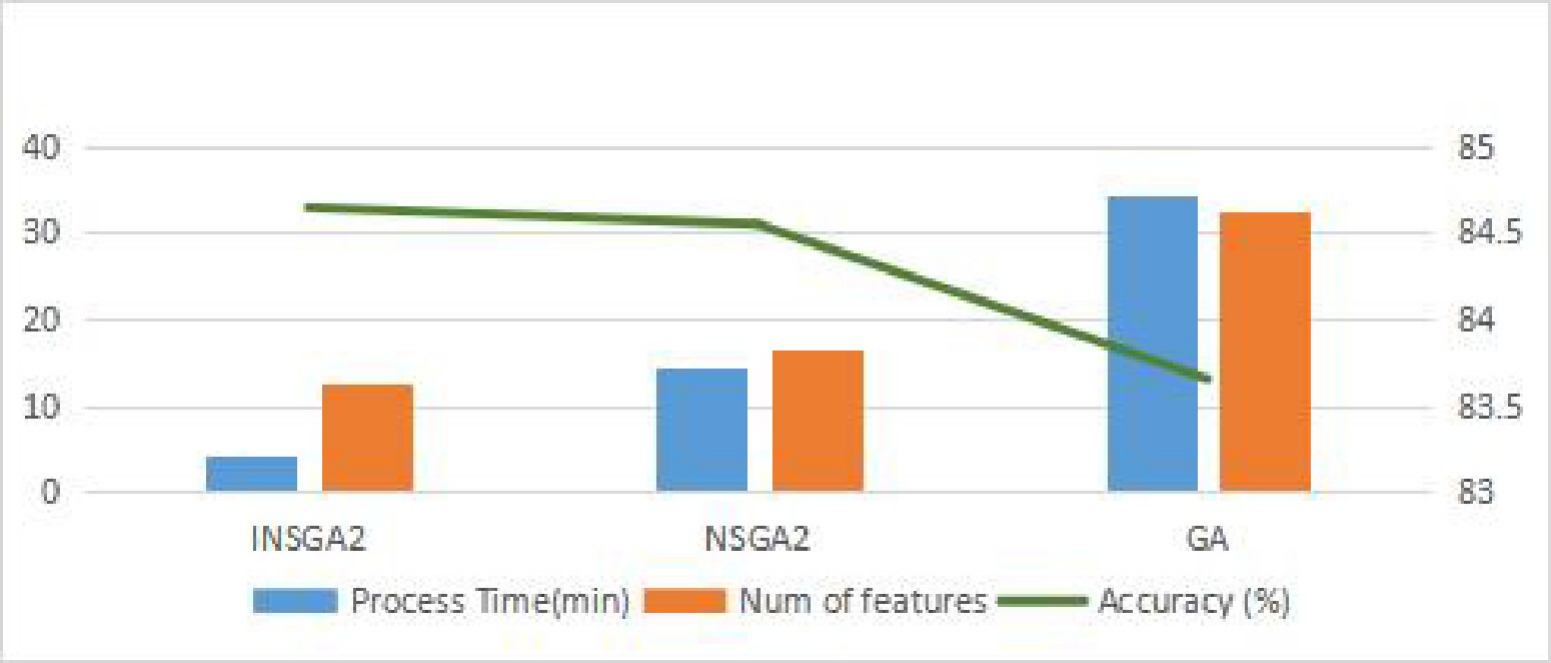
Comparison of average comprehensive evaluation of GA, NSGA-II and I-NSGA-II.

The average values of running time, number of selected features and accuracy of the experiments of the six data sets are shown in Table 8. The average accuracy of I-NSGA-II is slightly higher than that of the other two algorithms. However, I-NSGA-II has obvious advantages in the number of selected features and running time. Fig 11 shows the comparison of average comprehensive evaluation among objective, multi-objective and improved multi-objective.

**Table 8:** Comparison of average comprehensive evaluation of GA, NSGA-II and I-NSGA-II.

	Process Time(min)	Num of features	Accuracy (%)
I-NSGA-II	4.225725385	12.5	84.64333333

#### 4.8.3 Comparison of population initialization

**Fig 12:**
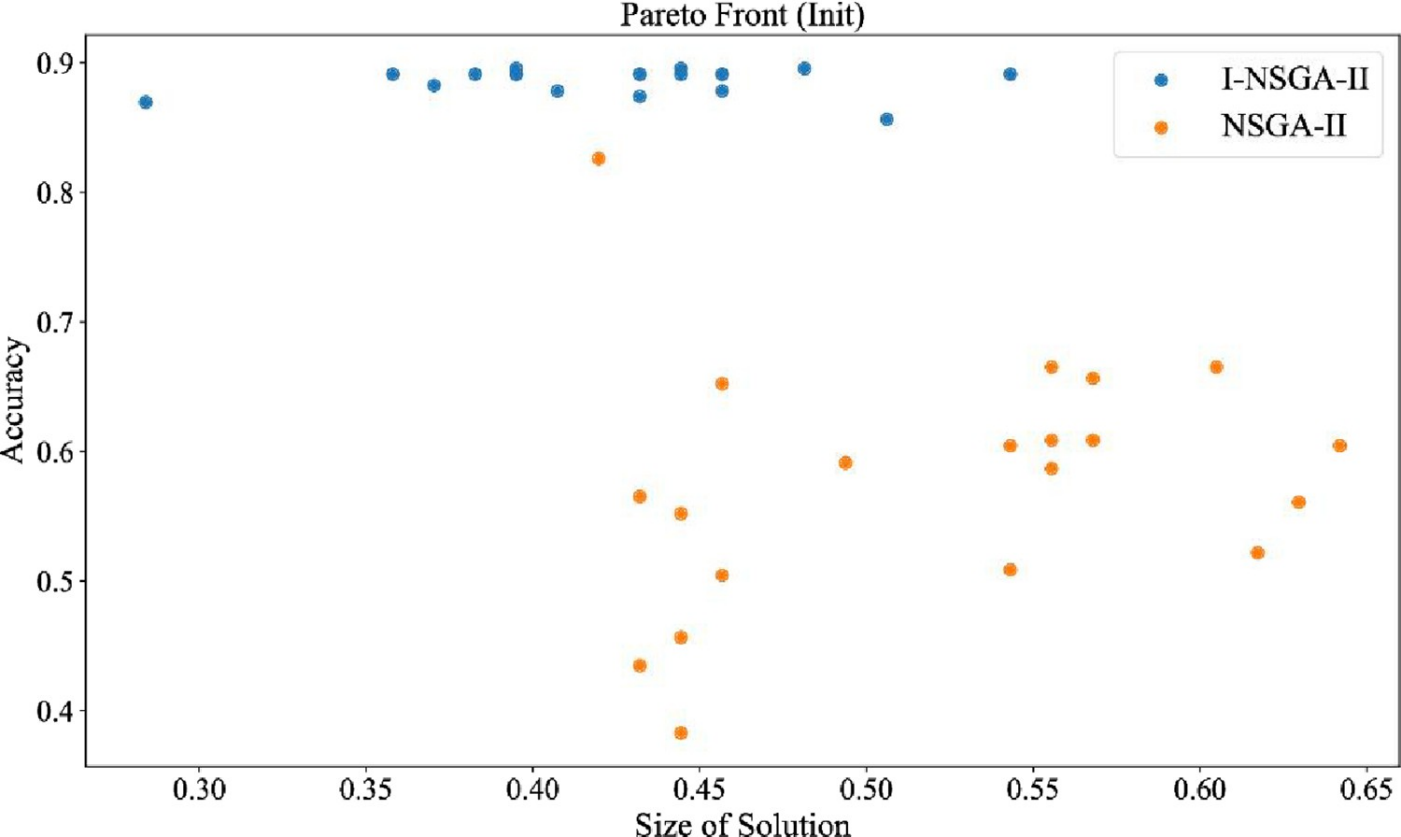
Population initialization comparison of I-NSGA-II and NSGA-II.

The I-NSGA-II algorithm proposed in this study adopts two different filtering feature selection methods to integrate information into a priori population in the population initialization stage. Introducing a priori information in the formal algorithm stage will help to promote the algorithm to find the global optimization. Fig 12 shows the initialization comparison between I-NSGA-II algorithm and NSGA-II algorithm. The horizontal axis solution size ratio is listed as the proportion of the selected feature number to the total feature number, that is, the first target represented by Eq (13). The vertical axis accuracy is the accuracy, that is, the second target represented by Eq (14). It can be seen from the Fig 14 that I-NSGA-II has a better solution than the original algorithm in the initial population (the closer to the upper left of the figure, the better).

### 4.9 Comparison with other models and algorithms

#### 4.9.1 Comparison of benchmark model

The algorithm proposed in this study is compared with the classical machine learning models including support vector machine, KNN, XGBoost, random forest (RF), decision tree (DT) and two neural network models with different layers commonly used in time series. Table 9 shows that I-NSGA-II has the highest accuracy, F1 score and AUC.

**Table 9:** Comparison of prediction performance of different methods.

	Accuracy (%)	F1-score (%)	AUC
I-NSGA-II-RF	**89.44**	**90.06**	**89.4**
SVM	85.65	87.55	84.99
KNN	79.57	82.78	78.47
XGBoost	62.17	71.66	59.33
RF	57.83	69.01	54.66
DT	49.57	49.57	50.11
ExtraTree	56.09	66.67	53.36
LSTM layer1	86.29	86.89	86.24
LSTM layer2	84.29	84.68	84.32
LSTM layer3	86.86	87.43	86.81
BiLSTM layer1	83.43	84.24	83.36
BiLSTM layer2	78.57	81.01	78.18
BiLSTM layer3	82.29	82.29	82.42

#### 4.9.2 Comparison of I-NSGA-II-RF and benchmark studies

I-NSGA-II-RF is compared with eight benchmark studies. The comparison models are based on LSTM, GA optimization, CNN, DNN and integrated learning. The comparisons are shown in Table 10. As for Hang Seng Index, I-NSGA-II-RF achieves the best performance except AUC. The average accuracy of I-NSGA-II-RF is the same as that of integrated learning algorithm. The average accuracy of both of them is better than those of the other six models.

**Table 10:** Comparison of I-NSGA-II-RF and benchmark studies.

Data sets	Author	Method	Accuracy	F1-score	AUC
Sentiment index +Apple (2013–2018)	(Jin, et al., 2020) [58]	S_EMDAM+LSTM	0.706	N/A	N/A
S&P500 index (2010–2017)	(Yang et al., 2020) [59]	CNN + LSTM	0.632	0.625	N/A
CITIC security stock (2017–2018)	(Long et al., 2020) [60]	CNN + BiLSTM	0.759	N/A	0.731
Tokyo Stock Exchange index (2007–2013)	(Qiu & Song, 2016) [61]	GA-ANN	0.813	N/A	N/A
KOSPI index (2000–2016)	(Chung & Shin, 2020) [3]	GA-CNN	0.738	N/A	N/A
NASDAQ index (2004–2015)	(Singh et al., 2017) [62]	(2D)^2^ PCA+ DNN	0.672	N/A	N/A
Bank of America (BAC) stock (2005–2019)	(Ampomah et al., 2020) [14]	Integrated learning	0.846	0.851	**0.928**
S&P500 index (2017–2017 intraday data)	(Sim et al., 2019) [63]	CNN	0.850	0.807	N/A
Hang Seng	this study	I-NSGA-II-RF	**0.894**	**0.901**	0.894
Average of six indexes	this study	I-NSGA-II-RF	0.846	0.855	0.845

#### 4.9.3 Comparison of recent studies that employ wrapper-based feature selection methods

We also compare recent studies that employ wrapper-based feature selection methods with our approach. The comparisons are shown in Table 11.

**Table 11:** Wrapper-based feature selection methods.

Study	Types of features	Feature selection	Prediction methods	Datasets	Prediction target	Performance	Evaluation metrics
Labiad et al. (2016) [64]	Technical indicators	RF	Gradient boosted trees (GBT), SVM, RF	Moroccan stock market	Direction of 10 min ahead	90%	Accuracy
Rana et al. (2019) [65]	Basic features	Decision tree classifier, Extra Tree classifier	LR, SVR, LSTM	Spanish stock market	Daily stock price	0.0151	RMSE
Nabi etal. (2019) [66]	Basic features	9 different methods	15 different classifiers	10 stocks from NASDAQ	Direction of monthly price	100%	Accuracy
Yuan et al. (2020) [67]	Technical indicators, Fundamental indicators	RFE, RF	SVM RF ANN	Chinese A-share stocks	Direction of excess returns	52%/53%	Accuracy, AUC
Iacomin (2015) [68]	Technical indicators	PCA GA	SVM	16 Forex stocks from Bloomberg	Direction of stock price	0.72	Accuracy
Das et al. (2019) [69]	Technical indicators	PCA, Factor analysis (FA), Firefly optimization (FO), Genetic algorithm (GA), FO with GA	ELM, OSELM, RBPNN	4 different stock market indices	Stock prices of 1,3,5,7,15,30 days in advance	143.1104 0.308 121.8011 0.0002543 0.0080	RMSE, MAPE, MAE, Theil’s U, ARV
Barak et al. (2017) [70]	Fundamental indicators	GA	Multiple classifiers	400 stocks	Return	83.6%	Accuracy
Farahani et al. (2021) [71]	Technical indicators	GA	ANN	5 stock indices	Close price	13.499	MAE

### 4.10 Comparison with deep learning

Long Short-Term Memory (LSTM) network is an improvement of RNN neural network, which have risen to prominence in the field of financial forecasting in recent years. Its special gate structure enables it to remember the long-term and short-term information of time series. LSTM and its hybrid forms are increasingly used in the stock market prediction studies [72]. It can handle the problems of RNN gradient disappearance and gradient explosion, so it has stronger prediction ability for complex nonlinear time series. BP, LSTM and BiLSTM are often used in time series prediction. The three kinds of neural networks are used as representatives to compare with the algorithm proposed in this study.

#### 4.10.1 Comparison with neural network

**Fig 13:**
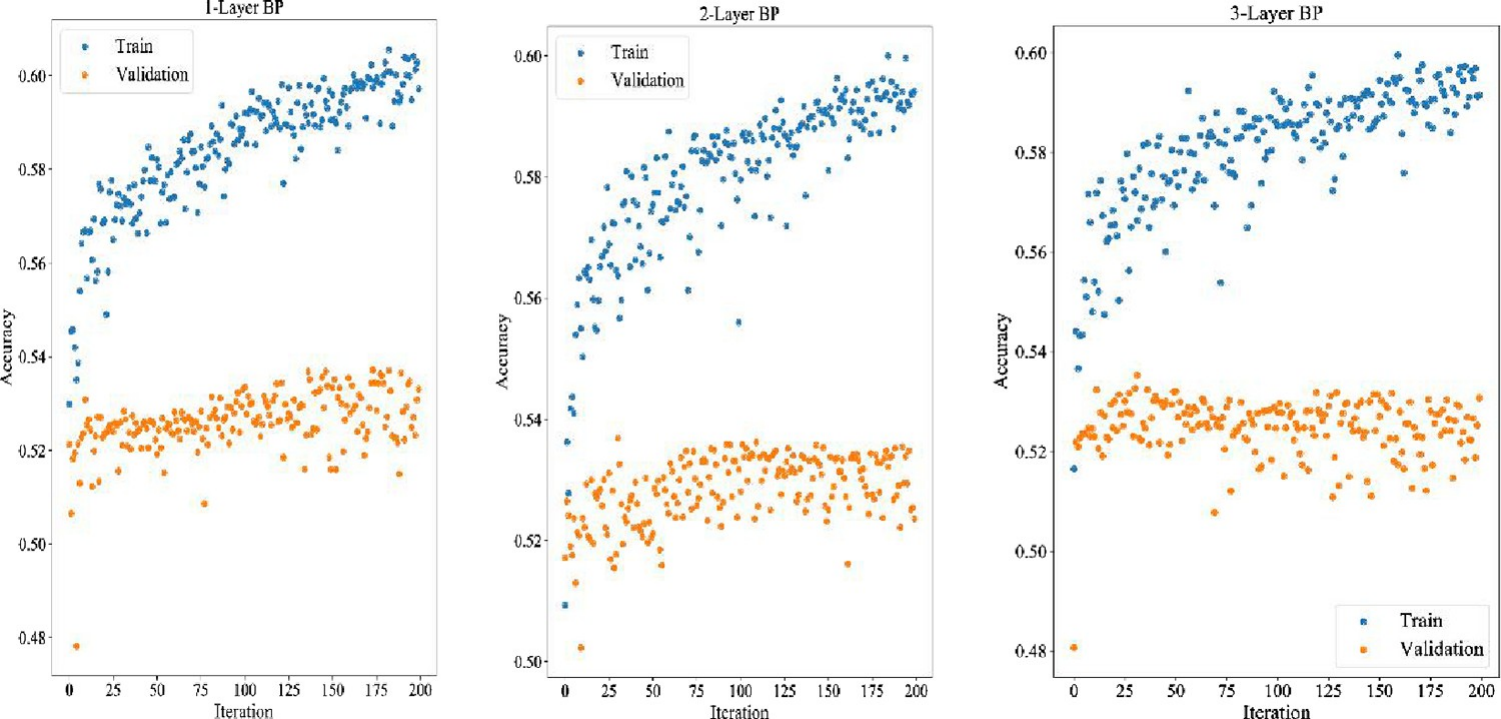
BP training and verification errors of different layers.

**Fig 14:**
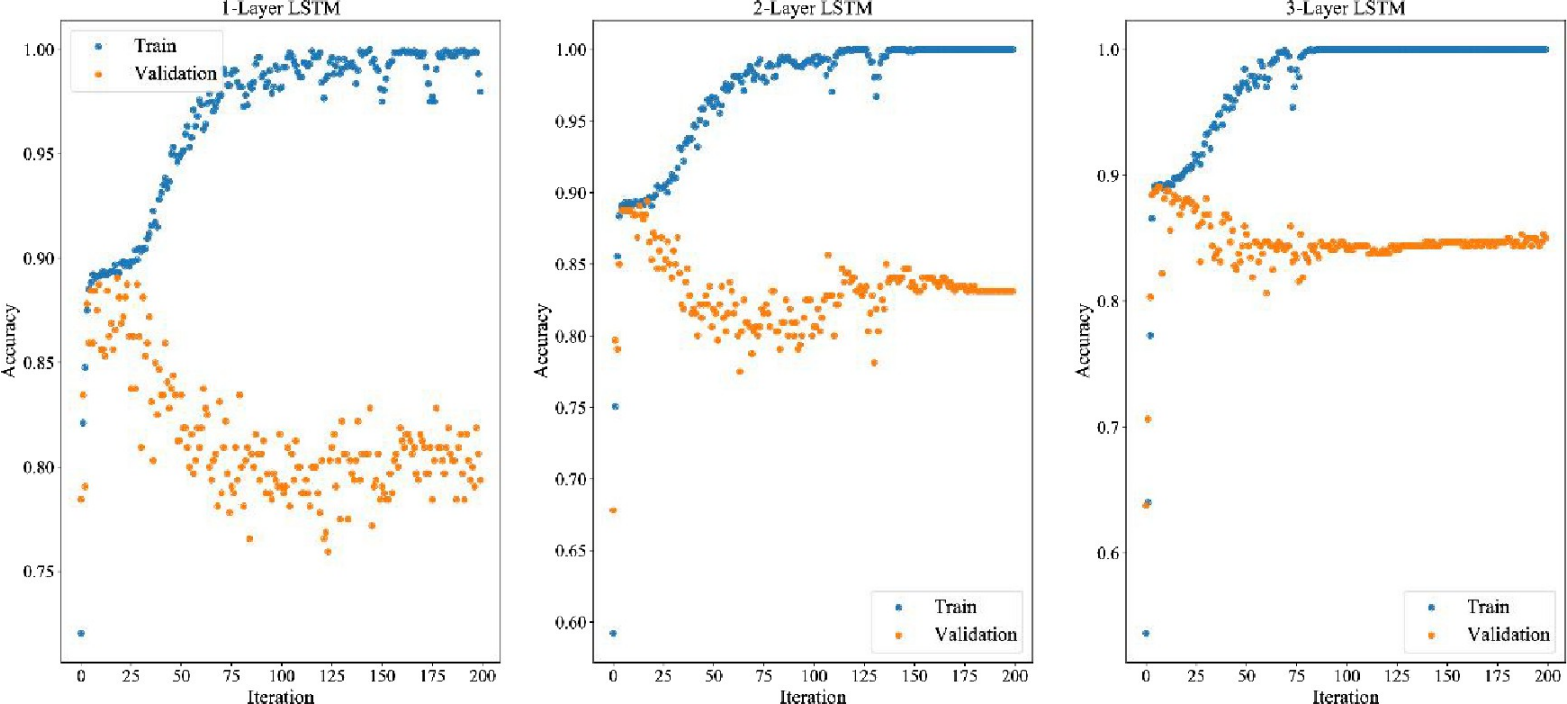
LSTM training and verification errors of different layers.

**Fig 15:**
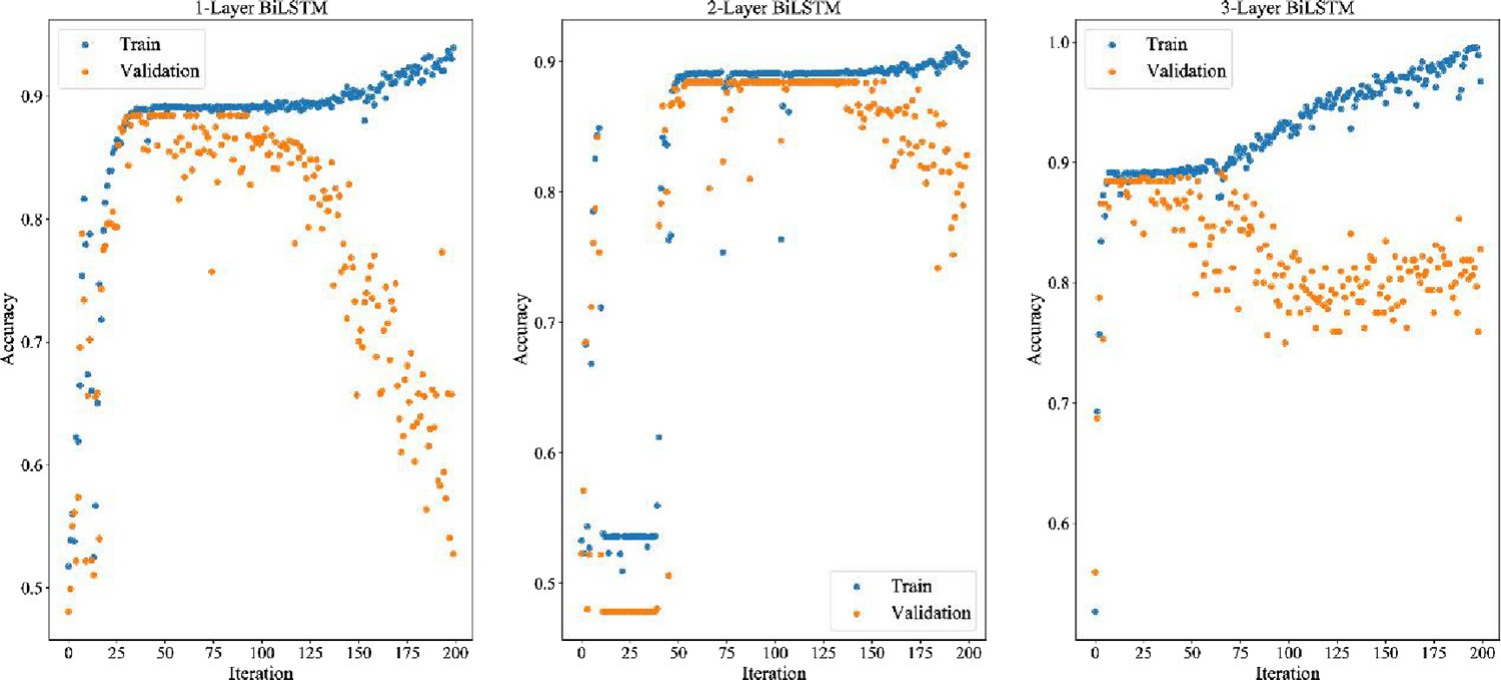
BiLSTM training and verification errors of different layers.

In section 4.10.1, the neural network is trained with default parameters, and the specific training parameters are shown in Table 12. In section 4.10.2, the neural network structure and training parameters will be adjusted by the TPE algorithm based on Bayesian optimization. All 81 features are used as inputs in both sections. The 80% of the inputs (the first 1560 trading days) are used as the training set, the 20% of the inputs (the next 390 trading days) are used as the test set. The last 20% of the inputs in the training set are used as the verification set. Each training is conducted 200 times. The training results are shown in Fig 13, Fig 14, Fig 15. Experimental results indicate that the structure of three-layer LSTM network has achieved the best effect among all the nine neural network structures. The verification accuracy is stable, which reaches 86.86%. The effect of BP neural network is the worst. The verification accuracy of its three structures has not reached 54% because it lacks memory structure. Therefore, it performs poorly in sequence prediction. The bi-directional memory structure of BiLSTM neural network has no obvious advantage over LSTM. The structure also causes the over fitting problem that the verification accuracy increases first and then decreases later. Due to the poor effect of BP neural network, we only show the experimental results of LSTM and BiLSTM in Table 9.

**Table 12:** Initial parameter setting of neural network.

	Optimizer	Layers	Nodes	Batch size
3-layer LSTM	Adam	(LSTM, LSTM, LSTM)	(100,64,32)	32
2-layer LSTM	Adam	(LSTM, LSTM)	(64,32)	32
1-layer LSTM	Adam	(LSTM)	(32)	32
3-layer BiLSTM	Adam	(BiLSTM, BiLSTM, BiLSTM)	(100,64,32)	32
2-layer BiLSTM	Adam	(BiLSTM, BiLSTM)	(64,32)	32
1-layer BiLSTM	Adam	(BiLSTM)	(32)	32
3-layer BP	SGD	(Dense, Dense, Dense)	(100,64,32)	32
2-layer BP	SGD	(Dense, Dense)	(64,32)	32
1-layer BP	SGD	(Dense)	(32)	32

#### 4.10.2 Comparison with LSTM neural network optimized by TPE

Deep learning is widely used in time series prediction because of its powerful prediction ability. However, the performance of the model largely depends on the structure of neural network and the adjustment of super parameters. Therefore, the number of network layers, the number of neurons in each layer and the forgetting rate in the super parameters need to be carefully adjusted to improve the accuracy of network prediction. In this section, TPE algorithm is used to adjust the structure of LSTM neural network, the number of nodes and super parameters. The specific ranges are as follows: the number of layers of LSTM neural network [1–3], the number of layers of dense neural network [1–3], the number of nodes per layer [32–128], and the forgetting rate [0.1–0.5]. The neural network structure is optimized by TPE algorithm for 200 times. A better structure is found in the optimization process, which is shown in Table 13. The best structure is that the three-layer LSTM connected to the three-layer Dense. The details are as follows: the number of network nodes is (115,110,72,57,92,44), the forgetting rate is 0.19, and the verification accuracy reaches 89.16%.

**Table 13:** Network structure optimized by TPE.

Structure	units	dropout	Accuracy (%)
LLDD	(97,60,55,78)	0.42	83.28
LLLDDD	(36,53,63,49,96,98)	0.46	83.9
LLDD	(124,83,90,33)	0.38	85.13
LLD	(88,99,33)	0.23	86.38
LLLDDD	(60,117,72,39,74,44)	0.32	86.99
LLLDDD	(115,110,72,57,92,44)	0.19	89.16

**Fig 16:**
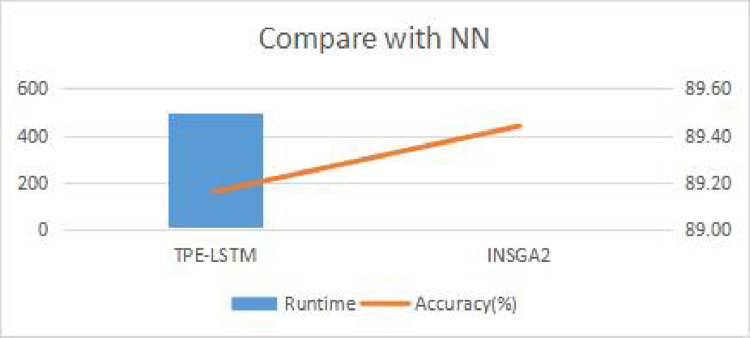
Comparison of I-NSGA-II-RF and TPE-LSTM.

Prediction performance and computing consumption are two important indicators to measure prediction model. The running time of the model is particularly important in the ultra short-term transaction. Therefore, the running time can better measure the performance of the algorithm compared with the calculation loss. TPE-LSTM algorithm has created 200 neural networks. Each neural network needs to be iterated 200 times. The operations are undoubtedly such a huge computing consumption that the running time will increase greatly. The running time and accuracy of I-NSGA-II-RF and TPE-LSTM algorithm are illustrated in Table 14. The accuracy difference between the two algorithms is very small. However, the running time of the algorithm proposed in this study is 0.85% of that of TPE-LSTM. Fig 16 visually shows the comparison of the running time and accuracy of the two algorithms.

**Table 14:** Comparison of I-NSGA-II-RF and TPE-LSTM.

	Runtime	Accuracy (%)
TPE-LSTM	497.1626526	89.16
I-NSGA-II-RF	4.225725385	89.44

### 4.11 Comparison with other multi-objective

**Fig 17:**
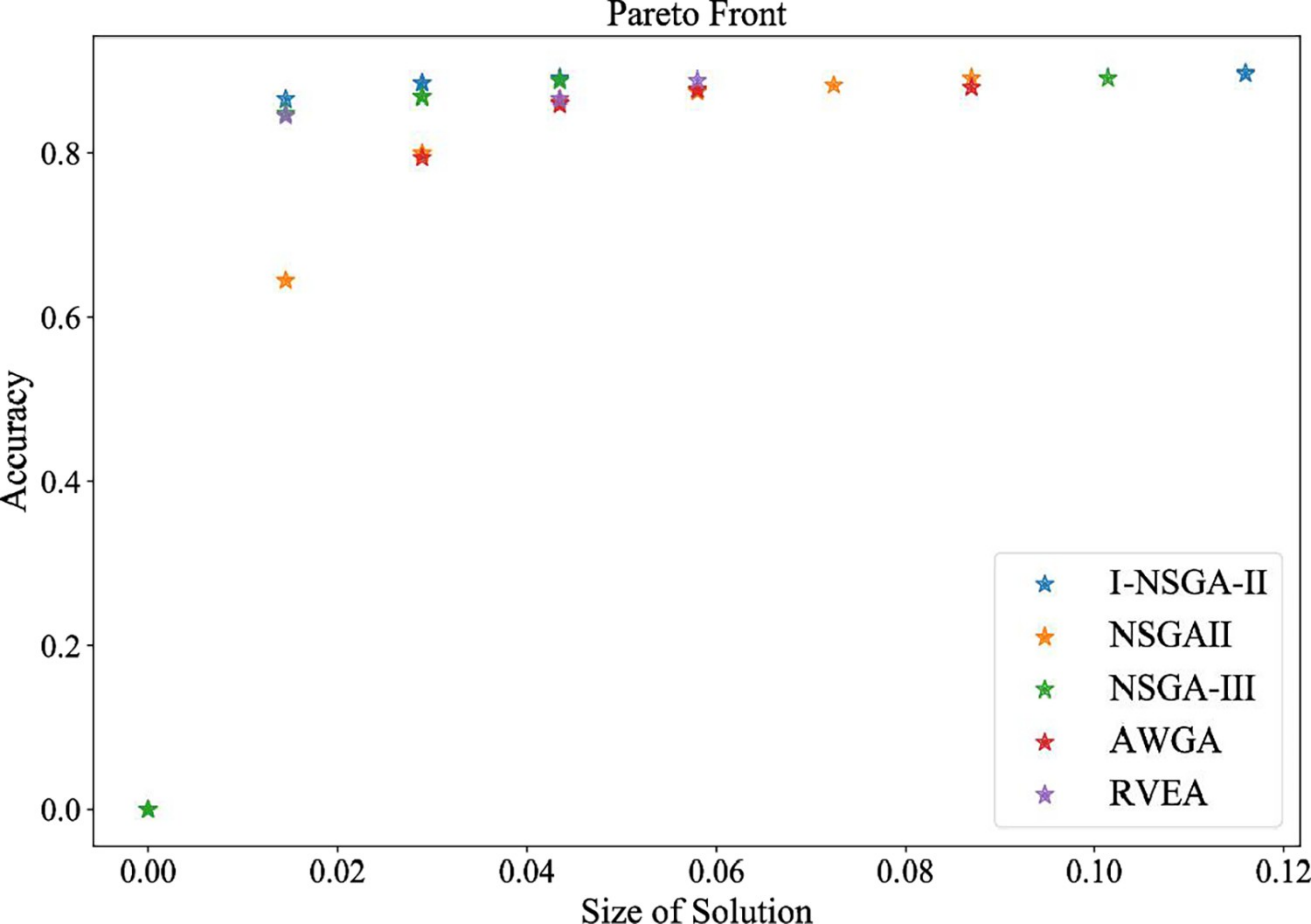
The pareto front obtained by each algorithm.

The I-NSGA-II-RF algorithm proposed in this study is compared with other multi-objective optimization algorithms. The experimental results of comparison of different multi-objective algorithms in Hang Seng data set are shown in Table 15 and Fig 17.

**Table 15:** Comparison of different multi-objective algorithms in Hang Seng data set.

	Num of features	Accuracy (%)	Non-dominated individuals
I-NSGA-II	8	**89.44**	200
NSGA-II	6	89.13	20
NSGA-III	7	89.11	20
AWGA	6	87.96	20
RVEA	4	88.83	20

### 4.12 Comparison with Wilcoxon’s rank-sum test and Friedman rank test

To further prove our model statistically in terms of significance, we conducted Wilcoxon’s rank-sum test and Friedman rank test (depending on the number of methods compared) to test the null hypothesis that there is no difference between the performance of our method and the baseline methods. We reported the p-values and effect sizes of the statistical tests and interpreted them according to the commonly accepted significance levels and guidelines. We compare the performance of the proposed method with the other methods (10 runs on each artificial dataset). The results shown in Table 16 are the p-values of the Friedman rand test and Wilcoxon signed-rank test. The differences between the proposed INSGA2-RF method and the NSGA2-RF, GA-RF methods are all significant at a level of 0.05 (95%).

**Table 16:** Non-parametric statistical tests on 6 artificial datasets.

Statistical test	NSGA2-RF	GA-RF
Sign-test	0.00222	0.01349
Wilcoxon signed-rank test	0.45637	0.00019

We conduct a Friedman rank test. The results mean the differences of three methods are all significant (statistic = 10.0, pvalue = 0.006737946999085468).

## 5. Conclusion

The algorithm I-NSGA-II-RF proposed in this study put forward the following contributions. (1) Significant novelty. Our scheme offers improvement strategies. First, I-NSGA-II-RF initializes the population by integrating two filtering feature selection methods. Then I-NSGA-II-RF regards the stock prediction problem as a multi-objective problem. Finally, I-NSGA-II-RF takes the solution of maximizing accuracy and minimizing resolution as the optimization direction to synchronously optimize the feature selection and the RF parameters. Compared with other benchmark studies, it has the fastest processing speed, the minimum solution and the highest prediction accuracy. (2) Good interpretability. There is a "black box" problem in DL methods as they cannot evaluate the importance of features. We put forward three-stage feature engineering. In the aspect of feature engineering, a total of 81 features including the original data and the expanded feature subsets are added, which improved the accuracy of the original prediction from 57.83% to 89.44%. The optimization effect is predominant. The problem of "Curse of dimension" is overcome reasonably. (3) Good performance. The prediction performance of I-NSGA-II-RF is the best among all the benchmark models. Compared with the original multi-objective algorithm and single objective algorithm, the running time of I-NSGA-II-RF is reduced by 70.85% respectively. The phase sum algorithm is 87.66% with high efficiency. Therefore, it is suitable for the prediction of short-term trading system. Compared with the deep learning algorithm with default parameters, the prediction performance is about 3% higher on average. I-NSGA-II-RF and TPE-LSTM with optimized parameters have the same accuracy, but the running time I-NSGA-II-RF is only 0.85% of that of TPE-LSTM.

These are some possible applications of the proposed method, but there may be more domains that can benefit from it. The proposed method of multi-objective optimization and feature engineering has some practical implications in other domains which are summarized as follows [73]. 1.Solving complex engineering problems that involve multiple conflicting criteria, such as design optimization, manufacturing, structural health monitoring, etc. 2. Enhancing the performance and efficiency of machine learning and data mining models that require feature selection and parameter tuning. 3. Finding trade-offs and compromise solutions for decision making in various fields, such as economics, agriculture, aviation, automotive, etc. In summary, the limitations and future work of our study are as follows. 1. Our study only focused on one specific domain of mechanical engineering problems and does not compare or generalize the proposed method to other domains or applications. In the future, we plan to apply our method to different types of engineering problems and evaluate its performance and applicability. 2. Our study did not provide a detailed analysis and explanation of the technical indicators used in the feature engineering process, which may result in some unreasonable or redundant indicators. 3.Our study did not provide enough details or explanations of the proposed I-NSGA-II-RF algorithm, such as its mathematical formulation, algorithmic steps, parameter settings, which may affect the algorithm’s stability and generalization ability. In the future, we plan to provide more technical details and illustrations of our algorithm and its implementation. 4. Our study only used six stock indices as our dataset, which may not be representative of all stock markets. In the future, we plan to design more experiments and conduct more analyses to validate and compare our method with existing methods.

## Supporting information

S1 File (RAR)

S1 Dataset (RAR)
